# Microbiota of the Pregnant Mouse: Characterization of the Bacterial Communities in the Oral Cavity, Lung, Intestine, and Vagina through Culture and DNA Sequencing

**DOI:** 10.1128/spectrum.01286-22

**Published:** 2022-08-02

**Authors:** Jonathan M. Greenberg, Roberto Romero, Andrew D. Winters, Jose Galaz, Valeria Garcia-Flores, Marcia Arenas-Hernandez, Jonathan Panzer, Zachary Shaffer, David J. Kracht, Nardhy Gomez-Lopez, Kevin R. Theis

**Affiliations:** a Perinatology Research Branch, Division of Obstetrics and Maternal-Fetal Medicine, Division of Intramural Research, Eunice Kennedy Shriver National Institute of Child Health and Human Development, National Institutes of Health, U.S. Department of Health and Human Services, Detroit, Michigan, USA; b Department of Obstetrics and Gynecology, Wayne State University School of Medicine, Detroit, Michigan, USA; c Department of Obstetrics and Gynecology, University of Michigan, Ann Arbor, Michigan, USA; d Department of Epidemiology and Biostatistics, Michigan State University, East Lansing, Michigan, USA; e Center for Molecular Medicine and Genetics, Wayne State University, Detroit, Michigan, USA; f Detroit Medical Center, Detroit, Michigan, USA; g Department of Biochemistry, Microbiology, and Immunology, Wayne State University School of Medicine, Detroit, Michigan, USA; h Department of Physiology, Wayne State University School of Medicine, Detroit, Michigan, USA; i Division of Obstetrics and Gynecology, School of Medicine, Faculty of Medicine, Pontificia Universidad Catolica de Chile, Santiago, Chile; University of Nebraska—Lincoln

**Keywords:** anoxic, atmosphere, cultivation, hypoxic, microbiome, mouse model, oxic, pregnancy, *Rodentibacter*

## Abstract

Mice are frequently used as animal models for mechanistic studies of infection and obstetrical disease, yet characterization of the murine microbiota during pregnancy is lacking. The objective of this study was to characterize the microbiotas of distinct body sites of the pregnant mouse—vagina, oral cavity, intestine, and lung—that harbor microorganisms that could potentially invade the murine amniotic cavity, thus leading to adverse pregnancy outcomes. The microbiotas of these body sites were characterized through anoxic, hypoxic, and oxic culture as well as through 16S rRNA gene sequencing. With the exception of the vagina, the cultured microbiotas of each body site varied by atmosphere, with the greatest diversity in the cultured microbiota appearing under anoxic conditions. Only cultures of the vagina were comprehensively representative of the microbiota observed through direct DNA sequencing of body site samples, primarily due to the predominance of two *Rodentibacter* strains. Identified as Rodentibacter pneumotropicus and Rodentibacter heylii, these isolates exhibited predominance patterns similar to those of Lactobacillus crispatus and Lactobacillus iners in the human vagina. Whole-genome sequencing of these *Rodentibacter* strains revealed shared genomic features, including the ability to degrade glycogen, an abundant polysaccharide in the vagina. In summary, we report body site-specific microbiotas in the pregnant mouse with potential ecological parallels to those of humans. Importantly, our findings indicate that the vaginal microbiotas of pregnant mice can be readily cultured, suggesting that mock vaginal microbiotas can be tractably generated and maintained for experimental manipulation in future mechanistic studies of host vaginal-microbiome interactions.

**IMPORTANCE** Mice are widely utilized as animal models of obstetrical complications; however, the characterization of the murine microbiota during pregnancy has been neglected. Microorganisms from the vagina, oral cavity, intestine, and lung have been found in the intra-amniotic space, where their presence threatens the progression of gestation. Here, we characterized the microbiotas of pregnant mice and established the appropriateness of culture in capturing the microbiota at each site. The high relative abundance of *Rodentibacter* observed in the vagina is similar to that of *Lactobacillus* in humans, suggesting potential ecological parallels. Importantly, we report that the vaginal microbiota of the pregnant mouse can be readily cultured under hypoxic conditions, demonstrating that mock microbial communities can be utilized to test the potential ecological parallels between microbiotas in human and murine pregnancy and to evaluate the relevance of the structure of these microbiotas for adverse pregnancy outcomes, especially intra-amniotic infection and preterm birth.

## INTRODUCTION

Ethical and practical limitations on experimentation with humans are barriers to fully understanding the role of the microbiome in human health and disease. To overcome these limitations, researchers often perform experiments with *in vitro* cell culture models or *in vivo* animal models, presuming that these models accurately reflect host-microbiome dynamics in humans. In particular, the laboratory mouse is widely used for *in vivo* experimentation evaluating microbial causes of disease (1, 2). The mouse model has several benefits. First, of the available mammalian models, mice are relatively inexpensive to maintain and easy to manipulate experimentally. They can be housed in controlled environments, including those that are germfree, thereby reducing the impact of potential confounding variables on the microbiota and experimental outcomes related to health and disease. However, the mouse model is often used without consideration of the differences between the microbiotas of mice and humans or the potential differential impacts of the microbiota on health and disease in the two species (1, 3, 4). Specifically, experimental mouse studies often include the introduction of a human-specific microorganism into the mouse’s microbiota or the transplantation of an entire body site-specific human microbiota into the mouse. A limitation of these studies is a lack of knowledge of a mouse’s typical microbiota, making experimentally induced changes in the microbiota hard to interpret. This is further exacerbated by studies operating under the assumption that the human microbiota can be equivalently recreated within the mouse model or that interactions between the human microbiota and a mouse are the same as those between the human microbiota and a human (2). However, if parallels between the microbial ecology of human and mouse body site-specific microbiotas can be identified, and if mouse microbiotas can be tractably constituted through culture, manipulated in a targeted way, and reintroduced into the mouse, then focusing on the mouse microbiota in investigations of mouse models of health and disease may be as or more fruitful than focusing on the human microbiota.

The intestinal microbiota of the mouse has been intensively studied and characterized (5–20). However, the microbiotas of the murine oral cavity, lung, and vagina have received much less attention (Tables S1 to 3), and only a few studies have simultaneously characterized the microbiotas of multiple body sites in the mouse (5, 18, 21). This gap in knowledge is particularly apparent in studies of the mouse microbiota during pregnancy. The mouse has been widely used to investigate pregnancy complications, including perinatal infection and preterm labor/birth (22–38); however, aside from the intestinal microbiota (15, 17, 39), the microbiota of the mouse in the context of pregnancy has been largely overlooked (Table 1). Given the widely reported associations between the human vaginal microbiota and pregnancy complications, such as intra-amniotic infection (40–43) and spontaneous preterm birth (36, 44–58), the baseline vaginal microbiota in the pregnant mouse should be thoroughly investigated and characterized. This is critical because the human vaginal microbiota is unique—humans are the only mammal known to have vaginal microbiotas that are often dominated by a single bacterial species (i.e., one of four *Lactobacillus* spp., principally Lactobacillus crispatus or L. iners and secondarily L. gasseri or L. jensenii) (59–61), and these microbiotas have been characterized into readily distinguishable vaginal community state types (CSTs) (62). These *Lactobacillus*-dominated CSTs (CSTs I to III and V) are generally perceived as being conducive to reproductive health. Conversely, the relationship between human reproductive health and the non-*Lactobacillus*-dominated and thus species-rich and diverse CST IV is more ambiguous (45, 55–57, 63–65). This disparity in health outcome, potentially related to the structure of the vaginal microbiota, is highlighted by the observation that women who do not have a *Lactobacillus*-dominated vaginal microbiota prior to or during early pregnancy typically transition to a vaginal microbiota of *Lactobacillus* dominance as gestation progresses (50, 51, 66), suggesting that pregnancy entails selective pressures for *Lactobacillus*-dominance in the vaginal microenvironment. Therefore, it is important to know if the vaginal microbiota of the mouse has characteristics similar or ecologically parallel to those of the human vagina microbiota, given the propensity for the use of the mouse model in studies of pregnancy, intrauterine infection, and preterm labor/birth.

**TABLE 1:** Description of previous 16S rRNA gene studies of the pregnant-mouse microbiome

Authors (reference)	Yr	Body site(s)	Microbiota culture methods	Mouse strain	Key microbiota findings
Gohir et al. (126)	2015	Intestine	Not performed	C57BL/6J	*Akkermansia*, *Clostridium*, *Bacteroides*, and *Bifidobacterium* were increased in pregnant control mice compared to nonpregnant mice. Other relatively abundant taxa observed in pregnant mice included *Lactobacillus*, *Alistipes*, and *Lachnospiraceae*.
Jašarević et al. (92)	2017	Intestine, vagina	Not performed	C57BL/6:129	Relatively abundant taxa in the intestine included S24-7, *Prevotella*, an unclassified *Clostridiales*, and *Sutterella.* In the vagina, the top mean relative abundant taxa were *Aggregatibacter*, *Lachnospiraceae*, and *Clostridiales* at embryonic day 7.5.
Nuriel-Ohayon et al. (127)	2019	Intestine	Not performed	Swiss Webster	Relatively abundant taxa included S24-7, *Clostridiales*, *Rikenellaceae*, *Bifidobacteria*, *Lachnospiraceae*, *Lactobacillus*, and *Turicibacter.*
Younge et al. (93)	2019	Intestine, vagina	Not performed	C57BL/6	The most relatively abundant taxa in the stool included S24-7, “*Candidatus* Arthromitus,” and *Allobaculum*, while the vagina was largely predominated by *Kurthia.*
Faas et al. (39)	2020	Intestine	Not performed	C57BL/6JOlaHsd	The intestinal microbiotas at gestational days 7 and 14 were similar to the microbiota before pregnancy; however, at gestational day 18, the microbiotas became less diverse and were predominated by *Allobaculum.*
Theis et al. (21)	2020	Intestine, lung, oral cavity, vagina	Homogenized tissue or Eswab fluid was plated onto tryptic soy agar with 5% sheep blood and chocolate agar plates under anoxic, hypoxic (5% CO_2_, 5% O_2_ and 90% N_2_), and oxic conditions at 37°C for 7 days.	C57BL/6	Several bacterial taxa relatively abundant in the intestine included S24-7, “*Candidatus* Arthromitus,” *Bacteroides*, *Helicobacter*, and *Lactobacillus*. The lung was predominantly S24-7 and *Lactobacillus*. The oral cavity was relatively abundant in Streptococcus, *Lactobacillus*, and Pasteurellaceae, while the vaginal microbiota was almost exclusively *Pasteurellaceae* and *Helicobacter*.
Liu et al. (128)	2021	Intestine	Not performed	ICR	Relatively abundant taxa in the control group included *Prevotellaceae* and Acinetobacter and were primarily composed of members of *Firmicutes* and *Bacteroidetes*.

The microbiotas of the oral cavity, lung, and intestine can also influence human pregnancy outcomes. Several studies have detected microorganisms from the oral cavities of pregnant women, especially Fusobacterium nucleatum, in the amniotic cavity, which is presumably due to hematogenous transfer and can result in stillbirth or spontaneous preterm birth (67, 68). Mycoplasma pneumoniae and Mycobacterium tuberculosis, bacteria known to colonize the human lung, have also been implicated in human intra-amniotic infection (69, 70). Additionally, Streptococcus agalactiae is a commensal bacterium in the human intestine and vagina; however, colonization of the neonate by this bacterium during delivery can cause adverse neonatal outcomes, such as sepsis (71–73). Given the potential for pregnancy complications caused in part by microorganisms from the oral cavity, lung, intestine, and vagina in humans, understanding the structure of these murine microbiotas during gestation is required if the mouse is to be effectively used as a model for investigating the role of the microbiota in obstetrical complications.

**FIG 1:**
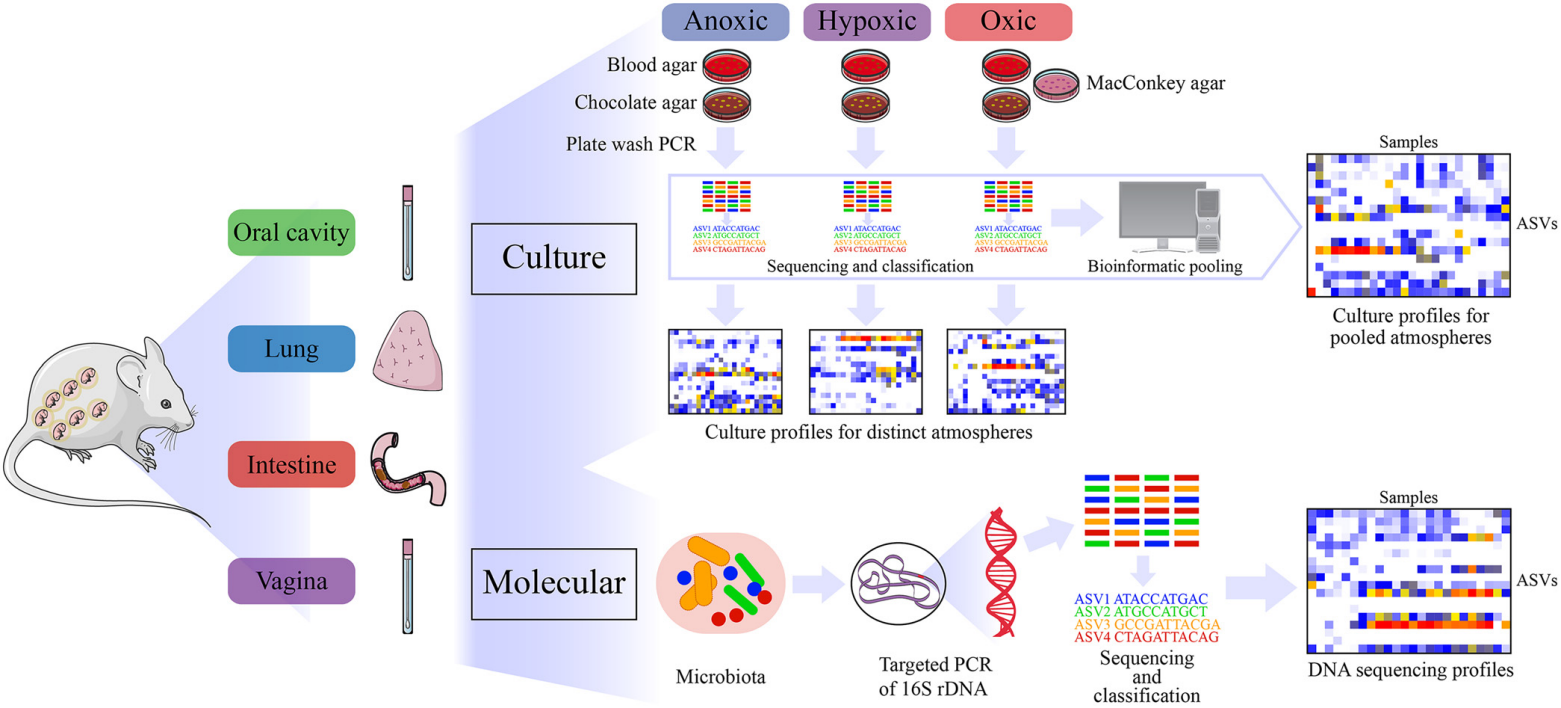
Study design for characterizing the microbiotas of the oral cavity, intestine, lung, and vagina of pregnant mice. Briefly, two sets of samples were collected from each body site of 11 pregnant mice. One set of samples was used for culture and the other for molecular surveys. Cultures were performed on samples from each body site, under three different atmospheric conditions on multiple medium types. Bacterial growth from each plate type was collected by plate washing with sterile PBS and then combined under each atmosphere. These samples subsequently had their DNA extracted followed by 16S rRNA gene amplification and sequencing. After classification of 16S rRNA gene sequences through DADA2, culture profiles for each body site under each atmosphere were generated as well as overall body site culture profiles after pooling of the sequence data from all three atmospheres. Samples for molecular surveys had their DNA extracted directly from the samples followed by 16S rRNA gene amplification, sequencing, and classification to generate molecular profiles. ASV, amplicon sequence variant; DADA2, divisive amplicon denoising algorithm 2; PCR, polymerase chain reaction.

Therefore, the objectives of this study were to characterize the microbiotas of the oral cavity, lung, intestine, and vagina of the pregnant mouse by using anoxic, hypoxic, and oxic culture, as well as 16S rRNA gene sequencing, and to compare and contrast the effectiveness of these different microbiological approaches for characterizing the mouse microbiota (Fig. 1). We found variation by atmosphere in the composition of microorganisms cultured, with a greater diversity of bacteria recovered under anoxic conditions. However, it was the profiles of bacterial communities cultured under hypoxic and oxic conditions that best matched the structure of the 16S rRNA gene profiles of sampled body sites. Each body site had a unique microbiota; however, multiple taxa were shared across body sites, suggesting a degree of interconnectedness among the microbiotas at these sites. Notably, potentially analogous to the human vaginal microbiota, the microbiota of the pregnant mouse vagina clustered primarily into groups based on the predominance of two congeners, Rodentibacter pneumotropicus and Rodentibacter heylii. Whole-genome sequencing of cultured isolates of these two *Rodentibacter* species revealed genes associated with the utilization of glycogen, the predominant carbohydrate in the vagina. Importantly, the profiles of bacterial communities cultured from the vagina tightly overlapped the 16S rRNA gene profiles of this body site. Hence, culture is likely sufficient to characterize the microbiota of the pregnant mouse vagina, and this microbiota can be successfully cultured and maintained in the laboratory and tractably manipulated for experimental *in vivo* studies of the vaginal microbiota and its role in pregnancy complications.

## RESULTS

### Influence of atmosphere and body site on the microbiotas cultured from the oral cavity, lung, intestine, and vagina.

**FIG 2:**
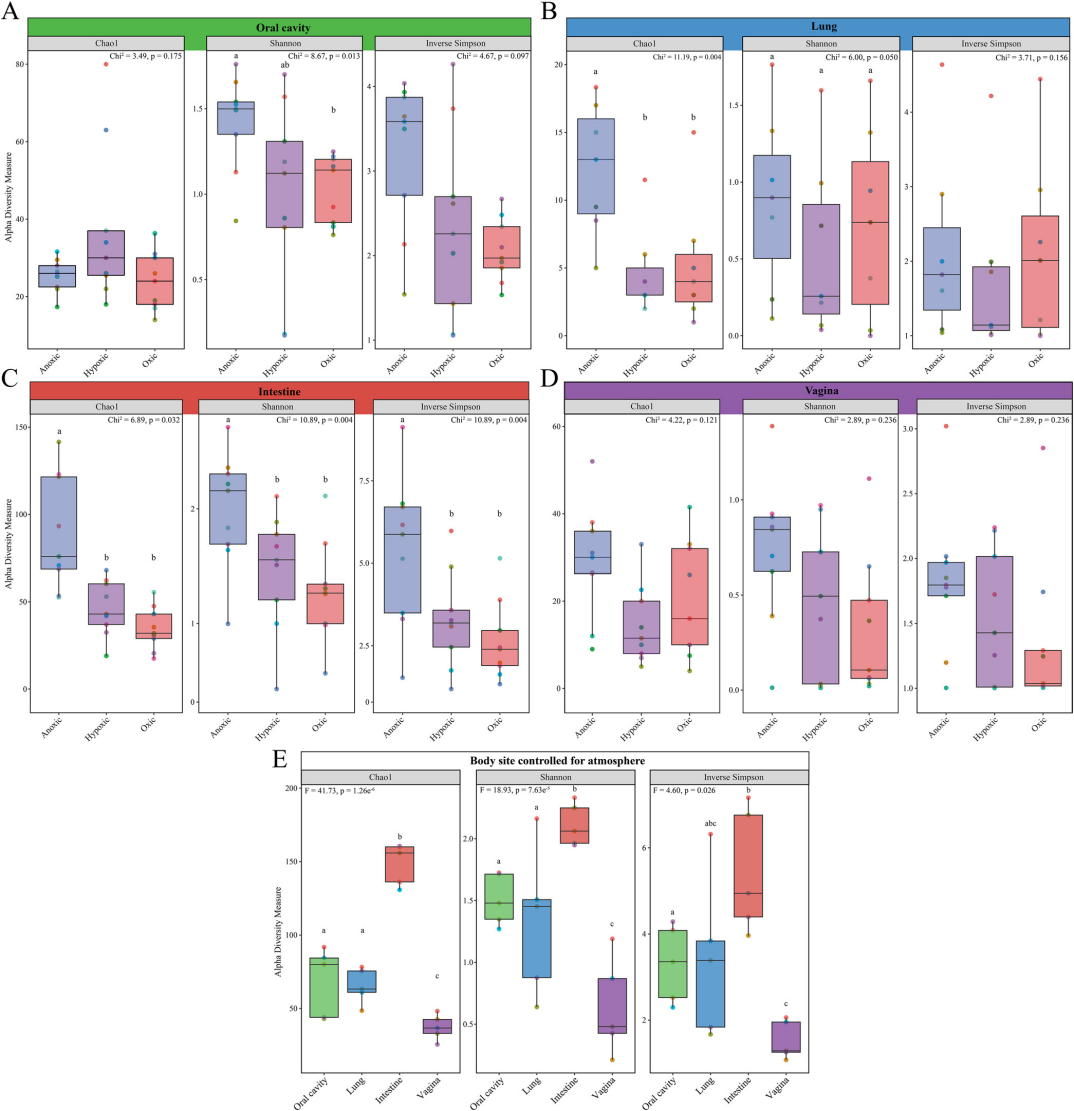
Alpha diversity comparisons between the microbiotas cultured under different atmospheres for the oral cavity, lung, intestine, and vagina and between body sites. Bar plots indicate differences in three alpha diversity measures among anoxic, hypoxic, and oxic cultures of the oral cavity (A), lung (B), intestine (C), and vagina (D) as well as across body sites (E). For panel E, culture data from each atmosphere for each individual mouse by body site were bioinformatically pooled, and only mice with culture data from all body sites and all atmospheres (*n* = 5) were included in the analyses. Data points are color coded by mouse ID and are consistent across panels. Lowercase letters that are shared within each panel indicate pairwise comparisons that were not significant.

**(i) Alpha diversity.** Alpha diversity (i.e., the diversity within a single community) of the cultured microbiota varied by atmosphere (i.e., anoxic, hypoxic, oxic conditions) in all body sites except the vagina (Fig. 2A to D). In general, the cultured microbiotas under anoxic conditions were more diverse than the cultured microbiotas under hypoxic and oxic conditions; this observation was most pronounced for the cultured intestinal microbiotas (Fig. 2C). After the cultured microbiota data from all atmospheres for each individual mouse by body site were bioinformatically pooled, variation in microbiota alpha diversity was clear among the four body sites (Fig. 2E). The cultured intestinal microbiota was consistently the most diverse, while the cultured vaginal microbiota was consistently the least diverse (Fig. 2E).

### (ii) Beta diversity.

**FIG 3:**
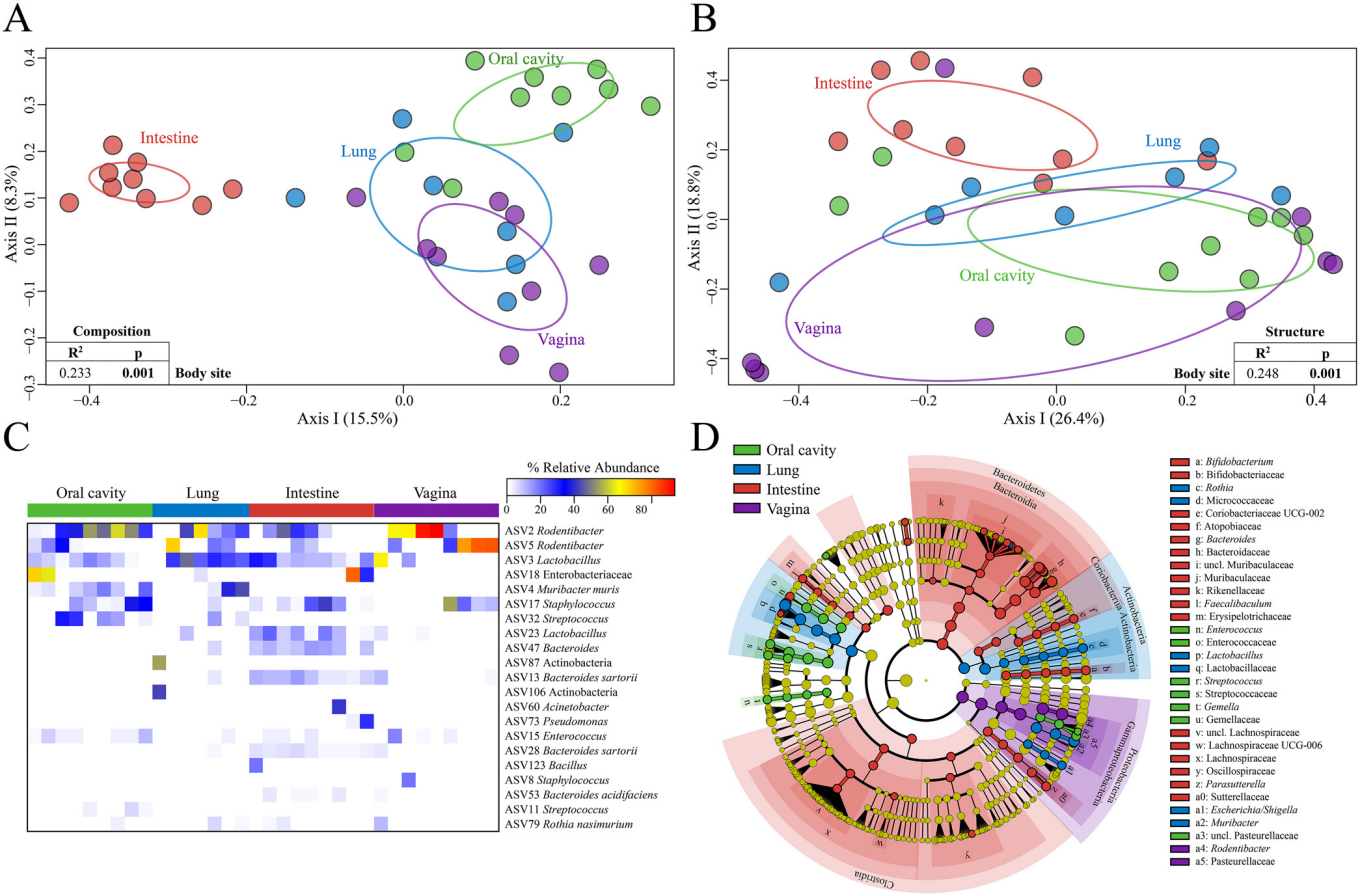
Comparisons of cultured microbiota from the oral cavity, lung, intestine, and vagina, controlled for atmosphere. (A and B) PCoA plots illustrating variation among cultured microbiota of the oral cavity, lung, intestine and vagina using the Jaccard dissimilarity index (A) for composition and the Bray-Curtis dissimilarity index (B) for structure. Ellipses indicate standard deviations. (C) Heatmap including ASVs with ≥1% average relative abundance within a single body site. Samples are clustered by Bray-Curtis similarities within each body site. (D) LEfSe analysis identifying taxa preferentially recovered from a particular body site. Each node represents a taxon at each taxonomic level starting with the kingdom *Bacteria* down through genus in the outermost nodes and are colored based on preferential recovery from a specific body site. Yellow nodes represent taxa that were not recovered preferentially from a particular body site. The diameter of each node is proportional to the relative abundance of that taxon. Phylum, class, and order (not labeled) clades are highlighted when significant for a particular atmosphere. ASV, amplicon sequence variant; LEfSe, linear discriminant analysis effect size.

Beta diversity (i.e., the diversity between two communities) of the cultured microbiota varied in composition and structure by both body site and atmosphere (Table 2; Fig. 3A and B; also, see Tables S4 to S6 in the supplemental material). Although atmosphere was a global driver of variation of the cultured microbiota, when the data for each body site were assessed separately by atmosphere (see Fig. S1 to 4), variation in microbiota composition and structure was not observed for the lung or vagina (Table S5; Fig. S2A and B and S4A and B). Notably, mouse identity contributed to variation of the bacteria cultured from the vagina but not to that of the bacteria cultured from the three other body sites (Table S5). Additionally, vaginal samples could be clustered into groups based on the most abundant cultured taxa (Fig. S4C). After bioinformatically pooling the culture data from all atmospheres by body site for each mouse, mouse identity and body site were identified as primary drivers of variation in the cultured microbiota (Tables 3 and 4; Fig. 3), suggesting that the different atmospheres may have masked the influence of mouse identity in the previous analyses. The clustering observed in the vaginal samples was still observed after pooling, suggesting the structures of the vaginal microbiota were largely independent of culture atmospheric conditions. Notably, the microbiotas of the vagina clustered into groups based on the relative abundance of either *Rodentibacter* or *Rodentibacter* co-occurring with Staphylococcus.

**TABLE 2:** Global comparisons of the cultured murine microbiota^*a*^

Beta diversity	Composition			Structure		
	F	*R* ^2^	*P*	F	*R* ^2^	*P*
Mouse ID	1.123	0.081	0.059	1.214	0.078	0.125
Body site	6.389	0.138	**0.001**	7.497	0.145	**0.001**
Atmosphere	2.541	0.037	**0.001**	5.576	0.072	**0.001**
ID * body site	0.986	0.199	0.556	1.231	0.222	0.058
ID * atmosphere	1.081	0.156	0.073	1.091	0.140	0.245
Body site * atmosphere	1.538	0.066	**0.001**	1.396	0.054	0.056

a Boldface indicates statistical significance. *Asterisk indicates the interaction between the two variables.

**TABLE 3:** Global comparisons of the cultured murine microbiota after bioinformatically pooling data across atmospheres by body site for each individual mouse^*a*^

Comparison	Composition			Structure		
	F	*R* ^2^	*P*	F	*R* ^2^	*P*
Mouse ID	1.348	0.314	**0.002**	2.056	0.395	**0.001**
Body site	3.171	0.221	**0.001**	3.833	0.221	**0.001**
Body site, controlled for ID	3.044	0.233	**0.001**	3.290	0.248	**0.001**

a Boldface indicates statistical significance.

**TABLE 4:** Pairwise comparisons (controlled for mouse ID) of the cultured murine microbiota after bioinformatically pooling data across atmospheres by body site for each individual mouse^*a*^

Comparison	Oral cavity (*n* = 9)			Lung (*n* = 7)			Intestine (*n* = 9)			Vagina (*n* = 9)		
	F	*R* ^2^	*P*	F	*R* ^2^	*P*	F	*R* ^2^	*P*	F	*R* ^2^	*P*
Oral cavity				1.90	0.12	**0.016**	5.29	0.25	**0.004**	2.45	0.13	**0.004**
Lung	2.16	0.13	**0.016**				3.40	0.20	**0.016**	1.27	0.08	0.094
Intestine	5.28	0.25	**0.004**	3.60	0.20	**0.016**				4.10	0.20	**0.004**
Vagina	2.48	0.13	0.059	1.97	0.12	0.094	4.43	0.22	**0.008**			

a The values in the upper right region refer to composition, and those in the lower left region refer to structure. Boldface indicates statistical significance.

### Influence of atmosphere, controlled for body site, on the cultured microbiota.

**(i) Oral cavity microbiotas preferentially recovered under different atmospheres.** Under anoxic conditions, cultures of oral cavity microbiota appeared to cluster based on the relative abundance of either (i) *Lactobacillus* (amplicon sequence variant [ASV] 3), Muribacter muris (ASV 4), and Streptococcus (ASV 32), or 2) *Rodentibacter* (ASV 2) and Staphylococcus (ASV 17) (Fig. S1C; Fig. 3C). This was contrasted with the cultures recovered under hypoxic and oxic conditions, which were consistently dominated by Muribacter muris, *Rodentibacter*, and Staphylococcus (Fig. S1C). Two linear discriminant analysis effect size (LEfSe) analyses were performed, one that was not restricted to a particular taxonomic classification level (i.e., hierarchical analysis) and one that was restricted to the level of ASV. Hierarchical LEfSe analysis revealed preferential recovery of bacteria from the phylum *Firmicutes* under anoxic conditions, specifically of the genera *Enterococcus*, *Lactobacillus*, and Streptococcus (Fig. S1D), while members of the phyla *Proteobacteria* and *Actinobacteria* were preferentially recovered under oxic conditions, including the genera *Rodentibacter* and *Rothia.* Specific ASVs of each of these genera were identified in the ASV-level analysis (Fig. S5A) and included prominent ASVs 2, 3, 15, and 79 (Fig. S1C).

### (ii) Intestinal microbiotas preferentially recovered under different atmospheres.

The microbiotas cultured from the intestine under anoxic conditions were characterized by high relative abundances of several *Bacteroides* and *Lactobacillus* ASVs as well as low relative abundances of *Bifidobacterium* and *Parasutterella* ASVs (Fig. S3C). Hierarchical LEfSe analysis revealed a large number of taxa that were cultured preferentially under anoxic conditions compared to the other atmospheric conditions (Fig. S3D). Notably, the phyla *Bacteroidetes* and *Actinobacteria* were heavily represented, as well as members of *Firmicutes*, especially *Lachnospiraceae* and *Oscillospiraceae*, and to a lesser extent members of the phyla “*Desulfobacterota* phyl. nov.” (74) (originally classified under the delta subdivision of *Proteobacteria*) and *Verrucomicrobia*. At the genus level, 13 genera were preferentially recovered in culture under anoxic conditions, including *Akkermansia*, *Bacteroides*, *Bifidobacterium*, *Colidextribacter*, *Coriobacteriaceae* UCG-002, *Desulfovibrio*, *Enterorhabdus*, *Faecalibaculum*, *Parasutterella*, *Lachnoclostridium*, *Lachnospiraceae* UCG-006, *Muribaculum*, and *Rikenella*. Staphylococcus was the only genus that was preferentially recovered under oxic conditions, and no genera were preferentially recovered under hypoxic conditions (Fig. S3D). The trends in the hierarchical analysis were consistent with those in the analysis restricted to the ASV level. With respect to the intestine, 18 ASVs were preferentially recovered under anoxic conditions, including Akkermansia muciniphila, multiple *Bacteroides* ASVs, *Bifidobacterium*, *Lactobacillus*, and *Parasutterella* (Fig. S5C). *Bacteroides* and *Lactobacillus* were also recovered in culture under hypoxic and oxic atmospheric conditions, but *Bifidobacterium* and *Parasutterella* were not (Fig. S3C). *Rodentibacter* and Staphylococcus ASVs constituted a large proportion of the cultures obtained under hypoxic and oxic conditions, yet they were not recovered under anoxic conditions (Fig. S3C). The ASV-only analysis identified only one feature as discriminant of oxic and hypoxic cultures, a Staphylococcus (ASV 17) and Bacteroides acidifaciens (ASV 26), respectively (Fig. S5C).

### (iii) Lung and vaginal microbiotas were not preferentially recovered under different atmospheres.

The profiles of microbiotas cultured from the lung and vagina were not affected by atmosphere (Fig. S2 and 4). However, LEfSe analysis identified Streptococcus (ASV 32) and Bacteroides sartorii (ASV 28) as being preferentially recovered under anoxic conditions from the lung (Fig. S5B). No taxa or ASVs were identified as being differentially recovered based on atmospheric conditions from the vagina.

### Influence of body site, controlled for atmosphere, on the cultured microbiota.

Between the four body sites, there were a total of 33 prominent ASVs (defined as having an average relative abundance of ≥1% in at least one body site and atmosphere combination) (Fig. S1C to S4C). Five ASVs were prominent among all four body sites (Table S7). These five ASVs were classified as *Rodentibacter* (ASVs 2 and 5), *Lactobacillus* (ASV 3), Staphylococcus (ASV 17), and Rothia nasimurium (ASV 79). Twenty of the 33 ASVs were prominent in only one body site, typically either the intestine or lung, and limited to one or two samples at high relative abundance or present in multiple samples at a low relative abundance (Table S8).

*Rodentibacter* was cultured from nearly all vaginal samples and at high relative abundance, yet the presence and abundance of the two *Rodentibacter* ASVs differed among vaginal samples. Specifically, in most mice, only one of the two *Rodentibacter* ASVs was abundant (Fig. S4C). In a minority of mice, both *Rodentibacter* ASVs were abundant. This contrasted with cultures from the other body sites, in which ASV 5 was much less common and ASV 2 was limited to recovery under hypoxic or oxic conditions, except in a few oral samples (Fig. S1C to S4C).

The prominent *Lactobacillus* (ASV 3) was cultured from most intestinal and lung samples regardless of atmosphere, exclusively under anoxic conditions, from most of the oral samples and was highly abundant in only a single vaginal sample (the only vaginal sample without *Rodentibacter*). Staphylococcus (ASV 17) was commonly cultured from oral and intestinal samples but only rarely from lung samples. In the vagina, Staphylococcus (ASV 17) was exclusively cultured from samples that had an abundance of *Rodentibacter* ASV 5; it was detected only alongside *Rodentibacter* ASV 2 when ASV 5 was also abundant.

After bioinformatically pooling the culture data from each atmosphere by body site for each mouse, 21 ASVs were prominent in at least one body site (Fig. 3C). Three ASVs were prominent among all four body sites, with the *Rodentibacter* ASVs 2 and 5 having the greatest average relative abundance in vaginal cultures (36.1% and 33.2%, respectively) and ASV 3 (*Lactobacillus*) having the greatest average relative abundance in lung cultures (24.1%). Ten of these 21 ASVs were prominent in only one body site, with six being prominent only in intestinal cultures. Only one of the 21 prominent ASVs was exclusive to a single body site; ASV 106, an unclassified *Actinobacteria*, was unique to the lung.

LEfSe analysis revealed many taxa that were cultured preferentially from the intestine (Fig. 3D). Specifically, members of the phyla *Bacteroidetes* and *Firmicutes* were preferentially recovered in the intestine (Fig. 3D). At the genus level, six genera were preferentially recovered in cultures of the intestine, including *Bacteroides*, *Bifidobacterium*, *Coriobacteriaceae* UCG-002, *Faecalibaculum*, *Parasutterella*, and *Lachnospiraceae* UCG-006. Members of the phylum *Actinobacteria* and genera Escherichia*/Shigella*, *Lactobacillus*, *Muribacter*, and *Rothia* were preferentially recovered from the lung. *Enterococcus*, Streptococcus, and *Gemella* were preferentially recovered from the oral cavity, while *Rodentibacter* was preferentially recovered from the vagina (Fig. 3D).

### Cultured microbiota contrasted with molecular characterizations of the same samples.

**FIG 4:**
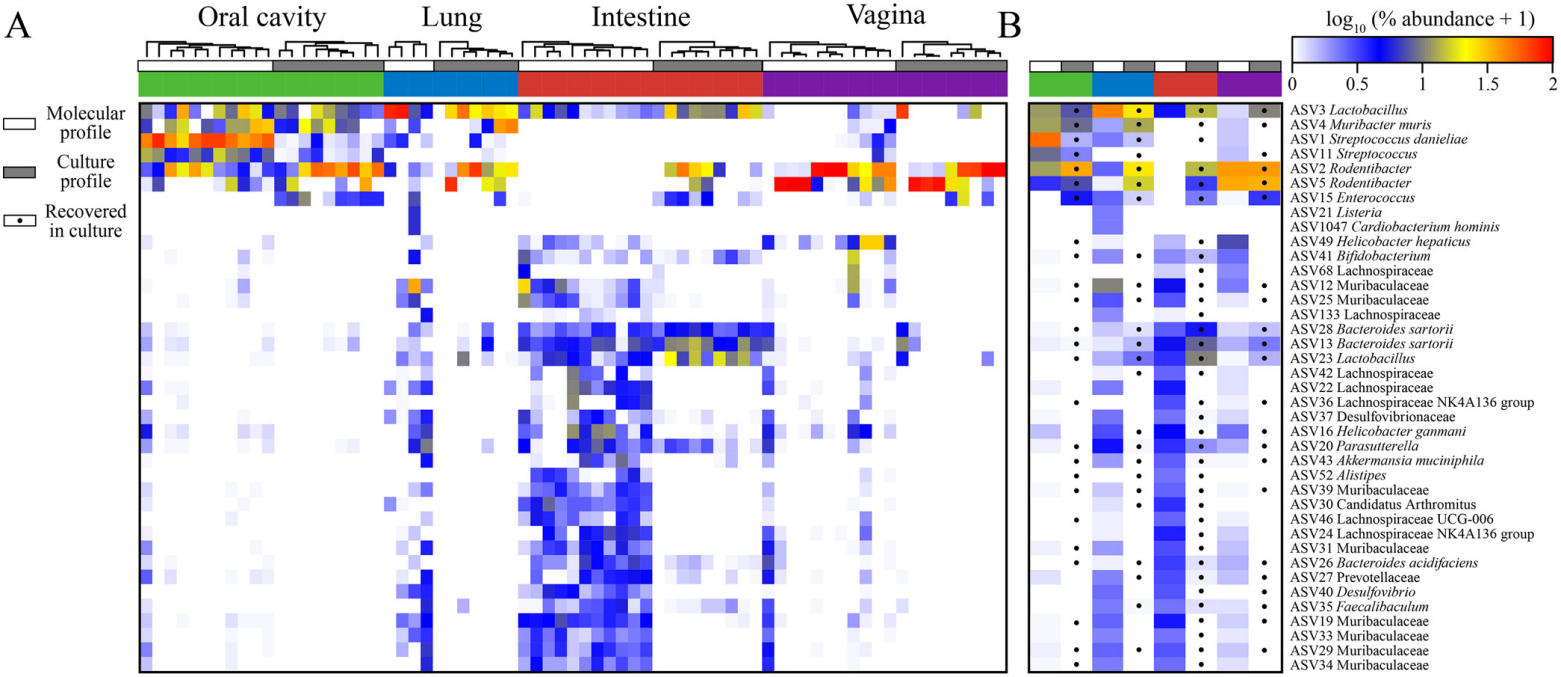
Comparisons of sequenced microbiota and cultured microbiota from the oral cavity, lung, intestine, and vagina. (A) Heatmap showing log-transformed percent relative abundances with hierarchical clustering based on Bray-Curtis values. (B) Molecular and culture profiles were separately averaged, with dots indicating whether an ASV was detected in culture. ASVs were included if they had a ≥1% average relative abundance in the molecular profiles for one of the four body sites. ASV, amplicon sequence variant.

Alpha diversity varied similarly for molecular profiles, as was observed in the cultured microbiotas. Variation was observed in both richness (Chao1; Friedman’s test: F = 16.91, *P* < 0.001) and evenness (Shannon and inverse Simpson, Friedman’s test: F = 16.91, *P* < 0.001) between the oral cavity, intestine, and vagina. Pairwise comparisons revealed that the intestine was more diverse than both the oral cavity (Wilcoxon signed-rank tests for all three indices: W = 66, *P* < 0.001) and vagina (Wilcoxon signed rank tests for all three indices: W = 66, *P* < 0.001), while the oral cavity and vagina were not (Wilcoxon signed rank test: Chao1, W = 18, *P* = 0.206; Shannon, W = 40, *P* = 0.577; inverse Simpson, W = 31, *P* = 0.898). The low alpha diversities were largely due to high relative abundances of Streptococcus danieliae (ASV 1) and *Rodentibacter* (ASVs 2 and 5) observed in the oral cavity and vagina, respectively (Fig. 4A).

In total, cultured surveys accounted for 411 ASVs, contrasted with 751 ASVs in molecular surveys (Table S9). Notably, only 339 ASVs were detected in both data sets; however, both data sets had numerous ASVs not observed in the opposing data set. For each body site, more ASVs were detected in molecular surveys than culture surveys except for the lung (Table S9). Of the prominent ASVs among both data sets (ASVs with an average relative abundance of ≥1% in at least one body site from culture or molecular samples), most ASVs were detected in both data sets overall, over 90% (46/51), and at least one-half were observed in both culture and molecular data sets at each body site (Fig. 4A). Only three of the 39 prominent molecular ASVs were not detected in culture surveys (Fig. 4B). Two of these were prominent only in the lung, while the third, ASV 22, was prominent in the lung and intestine and was detected in the intestines of all 11 mice. Of the 21 ASVs prominent in the cultured bacterial profiles (Fig. 4A), 11 were detected in all four body sites via molecular surveys while only two were not detected in any body site (ASVs 106 and 123). Despite sharing a majority of prominent ASVs, correlations between culture and molecular profiles were observed only among the intestine and vagina (Table 5), likely due to the overlap of prominent ASVs and the predominance of *Rodentibacter* ASVs in the vagina.

**TABLE 5:** Correlations between cultured microbiotas recovered under anoxic, hypoxic, and oxic atmospheres or after pooling of data from all three atmospheres and molecular 16S rRNA gene profiles^*a*^

Site	Anoxic		Hypoxic		Oxic		Pooled atmospheres	
	*r*	*P*	*r*	*P*	*r*	*P*	*r*	*P*
Oral cavity	−0.1185	0.7717	−0.5196	0.8655	−0.123	0.7409	−0.0662	0.6316
Intestine	0.4847	0.0616	0.3982	**0.0486**	0.6139	**0.0123**	0.5511	**0.0212**
Vagina	0.4564	**0.0072**	0.747	**0.0003**	0.7149	**0.0003**	0.6965	**0.0007**

a *r*, Spearman rank correlation coefficient. The lung could not be assessed due to low sample size. Boldface indicates statistical significance.

### Comparative genomics of the two predominant vaginal bacteria.

**FIG 5:**
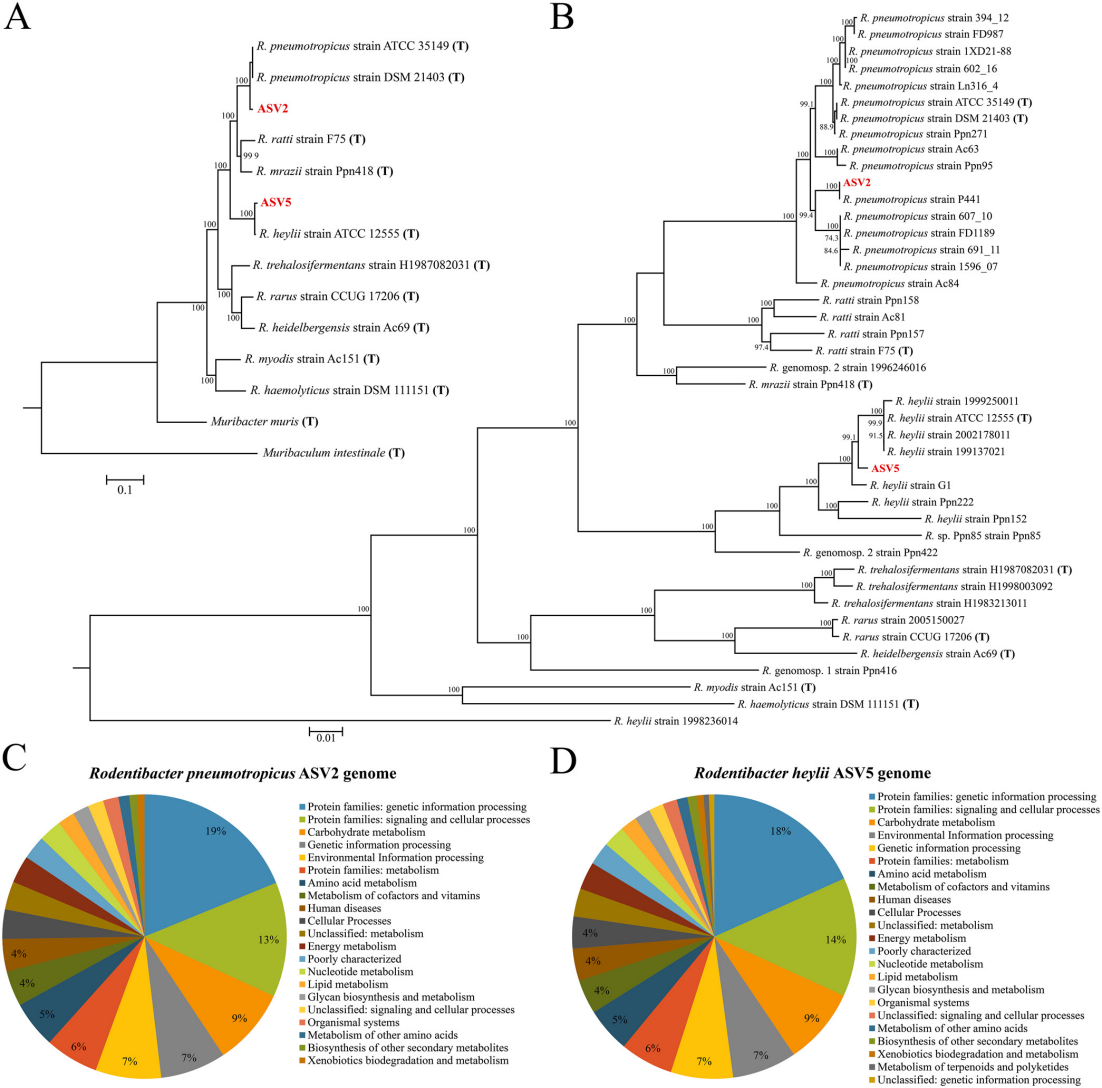
Phylogenomic and KEGG analysis of two vaginal *Rodentibacter* isolates. (A and B) Phylogenomic trees including the *Rodentibacter* isolates ASV 2 and ASV 5 and all *Rodentibacter* type strains (A) and all published *Rodentibacter* genomes. (C and D) Distribution of functional KEGG pathways enriched in the genomes of the two isolates. Phylogenomic trees were constructed by comparing 92 conserved bacterial genes as described by Na et al. (119). ASV, amplicon sequence variant; KEGG, Kyoto Encyclopedia of Genes and Genomes.

The distinct distribution and relative abundance patterns of ASV 2 and ASV 5 in the bacterial profiles of vaginal samples warranted further investigation of their genomic potential. ASV 2, identified as Rodentibacter pneumotropicus by 16S rRNA gene BLAST (75) analysis of sequenced isolates, and ASV 5, identified as Rodentibacter heylii, were submitted for whole-genome sequencing to assess how the genomic and functional features of these two distinct *Rodentibacter* isolates might explain their distribution and abundance patterns in the murine vagina. The assembled genomes of both isolates were incorporated into a phylogenomic analysis of *Rodentibacter* type strains (Fig. 5A), as well as all available *Rodentibacter* sp. genomes (Fig. 5B). The genomes of the ASV 2 and ASV 5 isolates clustered as expected, based on the 16S rRNA gene analysis, with the genomes of their conspecifics, and a summary of the general genomic features of the isolates and two additional strains is provided in Table S10.

Of the 2,384 genes present in the ASV 2 genome, 1,505 could be confidently assigned to a KEGG molecular network (76). The most represented categories were genetic information processing, environmental information processing, and carbohydrate metabolism (Fig. 5C). Analysis of complete pathways for carbohydrate degradation indicated that ASV 2 has the capacity to utilize glycogen (Fig. S6A and B) and 12 sugars: 2-deoxy-alpha-d-ribose-1-phosphate, d-arabinose, fructose, fucose, galactose, glucose, d-mannose, melibiose, ribose, trehalose, xylose, and nine-carbon keto sugars (sialic acids *N*-acetylneuraminate and *N*-acetylmannosamine). The genomic potential of ASV 2 was compared to that of 16 other reported Rodentibacter pneumotropicus strains for which published genomes were available. The published strains contained 1,565 core genes that were also present in ASV 2. Based on Prokka annotation of genomes (77), the pangenome of the 17 strains consisted of 4,389 genes, with each strain containing an average of 2,178 genes. Notably, ASV 2 contained the most genes (2,321) among these strains, followed by *R. pneumotropicus* strain Ac84 (2,311). Compared to the other *R. pneumotropicus* genomes, the genome of ASV 2 contained 83 unique genes, of which 81 are hypothetical proteins. The two unique genes with annotated functions were identified as DNA (cytosine-5)-methyltransferase (*ydiO*) and serine/threonine-protein phosphatase 1 (*pphA*). An additional 25 annotated genes were unique to ASV 2 and its most phylogenetically similar strain P441, including a secretory immunoglobulin A-binding protein (*esiB*), bifunctional polymyxin resistance protein (*arnA*), and lipooligosaccharide biosynthesis protein lex-1 (*lex1*).

For the *R. heylii* isolate ASV 5, 1,537 of 2,474 genes were confidently assigned to a KEGG molecular network (76), and as with ASV 2, genetic information processing, environmental information processing, and carbohydrate metabolism were the most represented categories (Fig. 5). Complete pathways for carbohydrate degradation were very similar to those for ASV 2, including glycogen metabolism (Fig. S6A and C), with the exception that ASV 5 is not able to degrade 2-deoxy-alpha-d-ribose-1-phosphate and it is able to degrade both l- and d-arabinose isomers, whereas ASV 2 can utilize only d-arabinose. The previously published genomes of seven *R. heylii* strains have a core genome of 1,649 genes, of which 1,644 were present in the genome of ASV 5. The five missing genes included those for two hypothetical proteins, lipopolysaccharide export system permease protein LptG (*lptG*), a duplicate outer membrane protein A (*ompA*), and a duplicate anthranilate synthase component 2 (*trpG*). Compared to the other *R. heylii* genomes, ASV 5 contained 182 unique genes, of which 155 were hypothetical proteins. Notable genes unique to ASV 5 include those for mRNA interferase toxin RelE (*relE*), a duplicate lysozyme RrrD (*rrrD*), very short patch repair protein (*vsr*), enterobactin exporter EntS (*entS*), a duplicate endoribonuclease ToxN (*toxN*) found in only one other strain, and colicin V secretion protein CvaA (*cvaA*). A unique feature of the genome of ASV 5 compared to those of other published *R. heylii* strains is the presence of genes from the *lsr* operon, which regulates the autoinducer-2 quorum sensing pathway, suggesting that this strain may exhibit quorum sensing, which may partially contribute to the distinct community structures observed in the present study.

Several differences in metabolic pathways were evident between the genomes of ASVs 2 and 5. As facultative anaerobes, the genomes of ASVs 2 and 5 include genes for fermentation; however, only ASV 2 has the necessary alcohol dehydrogenase gene, *adhE*, for metabolizing ethanol. Other features unique to ASV 2 include metabolism of nucleotide monophosphates, the amino acids alanine and proline, and the reduction of glutathione. Notably, ASV 2 is missing several enzymes involved in the tricarboxylic acid (TCA) cycle, including citrate synthase; conversely, ASV 5 is not. However, this observation was not unique to ASV 2, as these enzymes are also missing from the other published *R. pneumotropicus* genomes. Collectively, they encode citrate lyase, which is likely utilized as an alternative route for citrate degradation. Pathway features that are present in ASV 5 and yet missing in ASV 2 include lysine decarboxylase (needed for the biosynthesis of cadaverine), prepilin peptidase (involved in pilus formation), nitrite reductase (involved in denitrification), UDP-glucose:undecaprenyl-phosphate glucose-1-phosphate transferase (involved in colanic acid synthesis), and several enzymes necessary for the biosynthesis of the sialic acid CMP-*N*-acetylneuraminate. One interesting metabolic difference between the two ASVs is that ASV 5 contains two genes for the degradation of glycogen (Fig. S6A and C), whereas ASV 2 contains only one (Fig. S6A and B). Also, ASV 5 contains a suite of tight adherence protein genes (*tadB* and *tadD* to *-G*) and several, but not all, genes necessary for operation of the type IV secretion system; *virB2* and *virB7* were not identified in the genome. Last, although the genomes of both isolates contain the gene encoding the LuxS protein (a metabolic protein also utilized in quorum sensing), only isolate ASV 5 carries the necessary downstream genes for quorum sensing, suggesting a substantial ecological distinction between the two isolates.

Shared features of the genomes of both isolates involved in interacting with the extracellular environment include genes for the Sec-SRP and Tat export pathways, lap adhesins, type VI secretion system, and the metabolism of urea. Also, although the genome of ASV 2 does not have a putative prepilin peptidase gene, both isolates contain multiple genes involved in pilus formation. While both isolates share several notable functions associated with interacting and persisting in the environment, ASV 5 has a greater capacity to interact with the environment. The more robust genome of ASV 5 and the differences in metabolism warrant further exploration, as do the number of hypothetical proteins observed in the genomes of both species. Detailed experimental studies may elucidate the mechanisms underlying the distinct colonization patterns we observed in the mouse vagina, especially in the context of ASV 5’s apparent unique quorum-sensing ability.

## DISCUSSION

### Principal findings of the current study.

Preferential recovery of cultured microbiotas was observed between anoxic, hypoxic, and oxic atmospheres, with greater diversity of bacteria recovered under anaerobic conditions for each body site except for the vagina. The diversity of cultured microbiotas varied by body site, with the intestine having the highest and the vagina having the lowest bacterial diversity. While some variation was evident between the cultured microbiota and molecular surveys for each body site, there was a strong positive correlation between the cultured microbiotas and molecular profiles of the vagina. Bacterial profiles of the vagina were dominated by one or two distinct *Rodentibacter* strains (ASVs 2 and 5) while using both culture and molecular approaches, indicating that the culture approaches employed herein were representative of the vaginal microbiota. Whole-genome sequencing of these *Rodentibacter* strains identified many shared genomic features, including the ability to metabolize glycogen, yet there were also strain-specific features, most notably a suite of quorum sensing genes exclusively observed in the ASV 5 strain.

### Impacts of atmosphere on the cultured microbiota of the mouse.

Bacteria are capable of growth and reproduction in a variety of atmospheric conditions but are often broadly categorized by their ability or lack thereof to utilize O_2_ as a terminal electron acceptor during aerobic respiration under oxic conditions (78, 79). Notably, most of the body sites that were the focus of this study are typically low in O_2_ concentration compared to ambient atmospheres, thus often considered anaerobic environments (7). However, these sites exhibit an O_2_ gradient, as O_2_ diffuses out from the host tissues into the mucus layer and the tissue-microbiota interface (79). Therefore, it has been suggested that microbial culture at low O_2_ concentrations (i.e., hypoxic atmospheric conditions) will facilitate the growth of bacteria present at this interface, which are able to grow but are typically outcompeted by other bacteria at lower (anoxic) or higher (oxic) oxygen concentrations (i.e., the atmospheric conditions most frequently used for microbial culture) (79).

In the current study, anaerobic culture yielded the greatest diversity of bacteria for the intestine, lung, and oral cavity, but not for the vagina. This may suggest a bias of culturing anaerobic bacteria from the intestine, lung, and oral cavity or merely a greater capacity for anaerobic bacteria from these sites to grow under laboratory conditions. Regardless, the low degree of correlation between the culture and molecular profiles of the microbiotas in the oral cavity indicates that the culture methods used in this study were not sufficient for capturing the breadth of bacteria present in this body site. Notably, however, the microbiotas of the vagina that were cultured and subjected to molecular survey were largely congruent, especially when culture was performed under hypoxic conditions. This leads to two important conclusions. First, when culturing the vaginal microbiota of the pregnant mouse, culture under hypoxic conditions alone appears sufficient for capturing its members—oxic and anoxic cultures would need to be performed only if specific hypotheses about the microbiota and vaginal oxygen levels were being investigated. Second, the current study demonstrates that the vaginal microbiota of the pregnant mouse can be reliably captured through laboratory culture, thus it is feasible and tractable to generate culture libraries that can be used for *in vitro* and *in vivo* manipulative experimentation of the vaginal microbiota and/or intra-amniotic infection in murine animal models of pregnancy complications.

### Prior reports of the oral cavity, lung, and vaginal microbiotas of nonpregnant mice.

The microbiotas of body sites other than the intestine in laboratory mice have been only infrequently characterized by 16S rRNA gene sequencing. Studies characterizing the microbiotas of the oral cavity, lung, or vagina of normal nonpregnant mice are identified and summarized in Tables S1 to 3.

Most studies characterizing the microbiotas of the murine oral cavity have focused on a single mouse strain (i.e., C57BL/6) (Table S1). The genera within the oral microbiota often differed between studies, suggesting that environment plays a large role in the composition of the oral microbiota. This was demonstrated explicitly when the oral microbiotas of mice from different laboratories were compared (80). Of the relatively abundant genera in the oral cavity, *Lactobacillus*, Staphylococcus, and Streptococcus were observed in multiple studies (80–83). Notably, no studies have characterized the oral microbiotas of mice by using culture.

The microbiota of the murine lung has been characterized through several studies comparing the microbiota of diseased or treatment groups to that of control mice, as opposed to strictly descriptive studies of control or healthy mice (Table S2). Little overlap of abundant genera has been observed among studies. In fact, one study acquired mice from two different breeding facilities, characterized the microbiotas of the lungs, and found that there were no core bacteria common to all mice and not a single bacterium was shared between the majority of mice (84). However, the authors did observe convergence of the lung microbiotas of mice acquired from different facilities after a week of cohabitation, suggesting that the lung microbiota is dynamic and largely influenced by housing and social environments. Despite the pronounced role of the environment on the lung microbiota, several bacterial genera were relatively abundant in multiple studies: Streptococcus, *Lactobacillus*, Pseudomonas, and Staphylococcus (5, 84–87). Two studies of the lung microbiota utilized culture alongside molecular approaches. In the first, only one bacterium, *Micrococcus luteus*, was recovered and only from culture (5). In the second, *Stenotrophomonas* and *Ochrobactrum* were detected in both culture and molecular surveys of the lung (88).

Studies characterizing the vaginal microbiota of mice have also varied in the abundant genera observed; however, as with the microbiotas of the murine oral cavity and lung, members of *Lactobacillus*, Staphylococcus, and Streptococcus were observed in multiple studies (Table S3). Two studies each observed mice with similar vaginal microbiotas that could be clustered into at least two community state types (CSTs). In the first study, vaginal microbiota samples could be clustered into two CSTs based largely on the relative abundance of Streptococcus (>50% in one group and ≤10% in the other) (5). The second study included five vaginal CSTs, which were defined by varying relative abundances of Staphylococcus, *Enterococcus*, *Lactobacillus*, and multiple lower-abundance taxa (89). Although no study has characterized the vaginal microbiota in mice by using culture and molecular methods, two older studies did perform culture-based characterization of the vaginal microbiota in mice (90, 91). Both studies cultured members of Streptococcus, Staphylococcus, and *Lactobacillus*; one also consistently recovered *Corynebacterium* and *Actinomyces* (90), and the other recovered members of the families *Enterobacteriaceae* and *Bacteroidaceae* (91).

### Prior reports of the oral cavity, lung, intestinal, and vaginal microbiotas of pregnant mice.

Excluding our current and prior studies (21), the data from which overlap, the mouse intestinal microbiota during pregnancy has been characterized six times, and the vaginal microbiota during pregnancy has been characterized twice (Table 1). Among the studies that characterized the intestinal microbiota of pregnant mice (Table 1), approximately 18 bacterial taxa were observed at high relative abundances. The following taxa were observed at high relative abundances in multiple studies: S24-7, *Allobaculum*, *Bacteroides*, *Bifidobacterium*, “*Candidatus* Arthromitus,” *Clostridiales*, *Lactobacillus*, and *Lachnospiraceae*.

The two prior studies, which characterized the vaginal microbiota of pregnant mice, also simultaneously characterized that of the intestine (92, 93). In the first study, researchers investigated the effect of stress on these microbiotas and subsequent downstream effects on the microbial colonization of newborn mice. 16S rRNA gene sequencing was performed on maternal fecal samples collected daily and on vaginal fluid collected on embryonic day 7.5 (92). The bacterial taxa that were relatively abundant in the fecal samples included *Sutterella*, *Prevotella*, S24-7, *Bacteroides*, *Odoribacter*, *Desulfovibrionaceae*, *Lachnospiraceae*, *Ruminococcaceae*, and *Oscillospira*. The alpha diversity of the maternal intestinal microbiota decreased early in pregnancy, and the composition of the microbiota differed between early and late pregnancy. The vaginal microbiotas of the pregnant control mice at 7.5 days gestation were mainly composed of *Clostridiales*, *Aggregatibacter*, *Lachnospiraceae*, *Prevotella*, *Helicobacter*, and S24-7.

In the second study, researchers evaluated the fetal compartments of mice for evidence of *in utero* bacterial colonization and characterized maternal intestinal and vaginal microbiotas to assess the source of any potential bacterial signals detected in the fetus (93). Samples from the maternal stool were relatively abundant in “*Candidatus* Arthromitus,” S24-7, and *Lactobacillus*, while the vaginal samples were predominantly composed of Kurthia gibsonii.

In the current study, similar to prior studies (Table 1), we observed high relative abundances of *Muribaculaceae* (i.e., S24-7), *Bacteroides*, *Bifidobacterium*, *Desulfovibrionaceae*, *Lactobacillus*, and *Lachnospiraceae* in the intestinal microbiota of the pregnant mouse (Fig. 4). However, our findings for the vaginal microbiota were distinct. We found the vaginal microbiotas of pregnant mice to be dominated by *Rodentibacter*, *Helicobacter*, and *Lactobacillus* (Fig. 4). The differences between the microbiota observed by Jašarević et al. (92) and those described in the current study could be due to the gestational age at time of sampling. In the former, samples of the vaginal microbiota were taken during early gestation, embryonic day 7.5 (E7.5), whereas in the present study, they were taken at late gestation (E17.5), which may suggest a shift in the vaginal microbiota that occurs between early and late gestation. In the study by Younge et al. (93), vaginal samples were collected at one of three time points (E14 to -16, E17 to -18, and E19 to -20) between mid-gestation and late gestation; however, only two mice were sampled per group. The vaginal microbiotas of the earlier time point consisted primarily of “*Candidatus* Arthromitus” and S24-7, which was similar to what was observed in the stool samples from the same mice. At the latter two time points, the vaginal microbiotas were distinct from those of maternal stool, with low diversity and high relative abundance of Kurthia gibsonii. Although no *Kurthia* sequences were detected in the current study, the low diversity observed in both studies suggests that the murine vaginal microenvironment changes during pregnancy and is permissive to the dominance of certain bacteria in the vagina.

### Bacterial CSTs of the mouse vagina.

In a previous study, five CSTs were suggested for the nonpregnant mouse vagina (89). The authors described these as being dominated by Staphylococcus and/or *Enterococcus*, *Lactobacillus*, or a mixed population of bacteria. While these bacteria were also detected in our study of pregnant mice, aside from *Lactobacillus*, they were not observed at high relative abundances, suggesting a potential shift in the vaginal microbiotas of nonpregnant mice upon becoming pregnant. In our study, we almost exclusively observed a vaginal microbiota dominated by one or two distinct *Rodentibacter* strains that were widespread among the mice. These predominant strains/ASVs potentially mirror the dominance of Lactobacillus crispatus and *L*. *iners* in prominent CSTs of the human vagina (62), suggesting that the vagina of the pregnant mouse may represent a similar but unique ecological niche conducive to the proliferation of only a few predominant bacteria. This is especially interesting considering that during human pregnancy, the vaginal microbiota typically shifts even more dramatically to a *Lactobacillus*-dominated community, especially later in gestation and in women who had non-*Lactobacillus*-dominated communities before pregnancy (66).

### Novel insights into *Rodentibacter* strains in the pregnant mouse vagina.

The assembled genomes for cultured isolates of *Rodentibacter* ASVs 2 and 5 are representative of *R. pneumotropicus* and *R. heylii*, respectively (Fig. 5). It is unclear if these strains are uniquely adapted to murine vaginal microenvironments in general or if this phenomenon is limited to pregnant mice, or even potentially pregnant mice in the specific animal housing facility under investigation here. Notably, this may be a general phenomenon, as Jašarević et al. (94) recently found Pasteurella pneumotropica to be abundant in the vaginas of mice after pregnancy. In 2017, *P. pneumotropica* was reclassified to the genus *Rodentibacter* (95), indicating that this observation of *Rodentibacter* predominance is not exclusive to our animal facility.

The genus *Rodentibacter* (formerly Pasteurella pneumotropica) was first described in 2017 (95). *Rodentibacter* bacteria are Gram-negative rod-shaped microorganisms that are typically associated with laboratory and wild rodents. The first documented investigations of these bacteria were explorations of their disease-causing capabilities (96, 97). However, the pneumotropic *Pasteurella* bacteria, as originally described, were considered “latent” and rarely caused disease in colonized mice. The findings of the current study further indicate that asymptomatic colonization of pregnant mice by *Rodentibacter* is common and suggest the reconsideration of *Rodentibacter* species as commensal bacteria, especially in the murine vagina, with the potential to cause disease under certain circumstances.

The functional potential of both species suggests a wide range of metabolic capabilities, which may partially explain why both isolates were detected in multiple body sites of the mouse. In the original description (96) and subsequent reclassification as the genus *Rodentibacter* (95), these bacteria have generally been described as having the ability to metabolize a number of carbohydrates, including arabinose, dextrose, glycerol, inositol, lactose, maltose, mannose, sucrose, fructose, glucose, and galactose. Our genomic data are largely congruent with the experimentally documented metabolic capabilities of both *R. pneumotropicus* and *R. heylii*. Specifically, metabolism of fructose, fucose, galactose, glucose, mannose, melibiose, ribose, trehalose, and xylose has been demonstrated in both species and was identified in the genomes of our isolates. Notably, pathway analysis indicated that *R. pneumotropicus* lacked the genes necessary to metabolize l-arabinose while *R. heylii* did not, again consistent with the original characterizations of these species. Additionally, the genomes of both isolates indicate these strains can utilize glycogen (Fig. S6), a primary carbon source in the vagina (98). The larger genome size of ASV 5 and an extra copy of the gene for glycogen degradation may provide a more robust capacity to colonize and persist in the vaginal microenvironment and may partially explain why other ASVs co-occurred less frequently in ASV 5-dominated vaginal samples than in ASV 2-dominated samples. The consistencies between the known metabolic capabilities of these species and the genomic characterization of the two isolates in this study suggest the described pathways are in fact utilized by these bacteria; however, experimental validation of the metabolism (especially glycogen) of these specific strains is required.

The relationship of these two *Rodentibacter* isolates and their murine host appears highly similar to the relationship between Lactobacillus crispatus and *L*. *iners* and their human host. First, members of both genera are capable of inhabiting multiple body sites of their hosts (99–104). Second, *Rodentibacter* (96, 97, 104, 105) and *Lactobacillus* (106–108) species have been implicated in infections, suggesting similar relationships with their hosts in which, given the right environment and conditions, both genera are capable of causing disease. Third, the highest relative abundance among the populated body sites in both hosts is within the vagina, wherein relative abundances can exceed 90% of the sequenced microbiota (50, 59, 62, 66). Fourth, both isolates have the genomic capacity to degrade glycogen, a predominant carbon source in the mammalian vagina associated with the abundance of *Lactobacillus* in the human vagina (60, 109). Although *Rodentibacter* predominance in the pregnant mouse vagina has not been previously documented, this microbiota has been understudied (Table 1). It is possible that a low-diversity microbiota dominated by *Rodentibacter* is evidence of a shift in vaginal microbiota structure during pregnancy in the mouse. In humans, *Lactobacillus*-predominance during normal pregnancy is common and associated with healthy term gestations, whereas more diverse vaginal microbiotas are less common among pregnant women and have been associated with adverse pregnancy outcomes (60, 66). The *Rodentibacter*-dominated vaginal microbiotas observed in this study may represent a similar transition in mice. Specifically, the vaginal microbiotas of mice may be typically more diverse, akin to CST IV in the human vagina, and transition to a less diverse, *Rodentibacter*-dominated state during pregnancy. This needs to be investigated further.

### Strengths of this study.

This was the first study to simultaneously characterize the microbiotas of the oral cavity, lung, intestine, and vagina of the pregnant mouse through both culture- and 16S rRNA gene sequence-based approaches. It was also the first study to consider the extent to which culturing the microbiotas from these body sites under different atmospheric conditions captured the site-specific microbiotas, as defined through molecular surveys. This study revealed strong associations of *Rodentibacter* strains with the vagina of the pregnant mouse, and whole-genome sequencing of cultured representatives of these strains identified functional features that may explain their predominance with the murine vagina during pregnancy.

### Limitations of this study.

This study focused on C57BL/6 late-gestation pregnant mice from a single facility. This is important because there can be variation in the microbiotas of mice across facilities (110, 111) as well as in the same laboratories over time (19). Therefore, it is not yet clear the extent to which the patterns in body-site specific microbiota data reported herein can be extrapolated to other studies. Culture surveys were limited to plate washes of bacterial growth, so potential biases inherent in the growth of certain microorganisms over others in *in vitro* environments may have influenced the bacterial culture profiles of samples. This impacts the accuracy of evenness or heterogeneity measures of culture samples. Accounting for the variable growth between different bacteria is important in future work for capturing fastidious or rare microbiota via culture; however, the plate wash approach implemented here provided foundational knowledge of the typical culturable microbiota of the pregnant mouse. Characterization of the lung microbiota was constrained by sample size, most likely due to the low microbial biomass of this body site, resulting in poor-quality or low DNA sequencing numbers. Comparisons between molecular and culture surveys for this body site were limited; however, lung cultures were successful for only seven mice, potentially indicating that lung microbiotas may be transient or of very low biomass in some mice. Additionally, nonpregnant mice and samples from pregnant mice at different gestational ages were not included in this study, thus, the relationship of the microbiota throughout gestation could not be assessed. Nevertheless, this study provides detailed foundational knowledge, based on multiatmospheric culture and DNA-based sequencing approaches, of the microbiotas of the oral cavity, lung, intestine, and vagina of the pregnant mouse, thereby setting the stage for additional investigations into the reproductive microbial ecology of the mouse.

### Conclusions.

The microbiota of the pregnant mouse includes bacteria shared among the oral cavity, lung, intestine, and vagina. However, variation was evident in the microbiotas across body sites. Comparisons of culture and molecular microbiota profiles indicate that culture, especially hypoxic culture, largely captured the microbiota of the vagina but not necessarily that of the other body sites. Given that the predominant members of the vaginal microbiota can be effectively cultured in the laboratory, they can be tractably used for *in vitro* and *in vivo* experimentation evaluating relationships between the vaginal microbiota and adverse pregnancy outcomes in mice. The vaginal microbiota of the pregnant mouse appears to be dominated by one or two *Rodentibacter* strains, similar to the two *Lactobacillus*-dominated CSTs (i.e., I and III) in the human vagina during pregnancy. Whole-genome sequencing of the *Rodentibacter* strains dominating the pregnant-mouse vaginal microbiota here revealed the capacity to metabolize glycogen, a principal carbon source in the mammalian vagina. This capacity is also possessed by human vaginal lactobacilli. These findings suggest the existence of ecological parallels between the vaginal microbiotas of mice and humans during pregnancy. These parallels and their relevance to host reproduction warrant further investigation.

## MATERIALS AND METHODS

### Study subjects and sample collection.

Culture and DNA sequencing surveys of samples from the oral cavities, lungs, intestines, and vaginas of 11 pregnant mice included in our previous study evaluating the *in utero* colonization hypothesis (21) were analyzed here in depth in an effort to characterize and compare the composition and structure of the pregnant-mouse microbiotas across body sites (Fig. 1). These mice were C57BL/6 specific-pathogen-free (SPF) and were purchased from The Jackson Laboratory and bred in the SPF animal care facility at C.S. Mott Center for Human Growth and Development at Wayne State University, Detroit, MI, USA. This study includes previously unpublished information on the culture of microorganisms from these body sites across atmospheric and growth medium conditions as well as functional genomic information on the principal *Rodentibacter* species inhabiting the murine vagina. Animal procedures were approved by the Institutional Animal Care and Use Committee at Wayne State University (protocol 18-03-0584).

### Bacterial culture.

Bacterial culture was performed on intestinal and lung tissues and on oral and vaginal swabs under oxic, hypoxic (5% O_2_, 5% CO_2_), and anoxic (5% CO_2_, 10% H, 85% N) conditions at 37°C for 7 days. Under each atmosphere, samples were plated in duplicate onto tryptic soy agar with 5% sheep’s blood and chocolate agar. Samples were also plated on MacConkey agar under oxic conditions. If bacterial growth was observed (most typically a lawn of bacteria or too many colonies to count), the bacteria were collected by pipetting 1 to 2 mL of sterile phosphate-buffered saline (PBS) solution onto the agar plate and dislodging colonies with sterile and disposable spreaders and loops. These plate wash solutions (112) were stored at −80°C until DNA extractions were performed. DNA extractions were completed using a Qiagen (Germantown, MD) DNeasy PowerSoil extraction kit, as previously described (21). The V4 regions of the 16S rRNA gene copies in DNA extractions were targeted using protocols previously described by Kozich et al. (113) and sequenced on an Illumina MiSeq system at Wayne State University, as previously described by Theis et al. (21). Ultimately, 16S rRNA gene sequence libraries were generated for the cultures from 117/132 (89%) murine body site samples.

### DNA sequencing surveys.

Tissue samples of the lung, distal intestine, and proximal intestine were collected in addition to swabs of the oral cavity and vagina and stored at −80°C until DNA extractions were performed. DNA extractions of samples for molecular surveys were performed in a biological safety cabinet by study personnel donning sterile surgical gowns, masks, full hoods, and powder-free exam gloves. Extracted tissue masses ranged from 0.016 to 0.107 g, 0.053 to 0.097 g, and 0.034 to 0.138 g for the lung, distal intestine, and proximal intestine, respectively. Two types of negative technical controls were included in the DNA extraction and sequencing processes to address potential background DNA contamination: (i) sterile swabs as a negative control for body sites sampled with a swab (i.e., oral and vaginal sites) and (ii) extraction tubes with no biological input as a negative control for body sites from which tissue was collected (i.e., proximal intestine, distal intestine, and lung).

DNA was extracted from tissues, swabs, and technical controls (i.e., swabs [*n* = 11] and blank DNA extraction kits [*n* = 23]) by using the Qiagen DNeasy PowerLyzer PowerSoil kit with minor modifications to the manufacturer’s protocol. Specifically, samples were added to the supplied bead tube along with 400 μL of bead solution, 200 μL of phenol-chloroform-isoamyl alcohol (pH 7 to 8), and 60 μL of solution C1. Mechanical lysis of cells was done by using a bead beater for 30 s. Following centrifugation, the supernatants were transferred to new tubes, 100 μL of solutions C2 and C3 in addition to 1 μL of RNase A enzyme was added, and tubes were incubated for 5 min at 4°C. After centrifugation, supernatants were transferred to new tubes containing 650 μL of solution C4 and 650 μL of 100% ethanol prior to addition to the filter column and 60 μL of solution C6 for elution. The lysates were loaded onto filter columns until all sample lysates were spun through the filter columns. Five hundred microliters of solution C5 was added to the filter columns and centrifuged for 1 min, the flowthrough was discarded, and the tube was centrifuged for an additional 3 min as a dry spin. Finally, 100 μL of solution C6 was placed on the filter column and incubated for 5 min before centrifuging for 30 s to elute the extracted DNA. Purified DNA was stored at −20°C until 16S rRNA gene sequencing. Amplification and sequencing of the V4 region of the 16S rRNA gene were performed at the University of Michigan’s Center for Microbial Systems as previously described (21), with library builds performed in triplicate and pooled for each individual sample prior to the equimolar pooling of all sample libraries for multiplex sequencing.

### 16S rRNA gene sequence processing of bacterial culture and molecular samples.

To allow greater taxonomic resolution of the murine maternal microbiota, raw sequence reads were processed by using the DADA2 package in R and following the tutorial pipeline as described by Callahan et al. (114) with minor modifications. Specifically, the reverse-read truncation length was increased from 160 to 200 [“truncLen=c(200, 240)”], the maximum expected errors in reverse reads were increased from 2 to 7 [“maxEE=c(2, 7)”], and for sample inference, samples were pooled to increase sensitivity (“pool=TRUE”). Sequences were ultimately classified into amplicon sequence variants (ASVs) and taxonomically identified using the Silva rRNA database v 138.1 (115, 116). After processing of 16S rRNA gene sequences through DADA2, any ASVs identified as mitochondria or chloroplasts and those not assigned to a bacterial phylum were removed.

Following DADA2 processing, quality filtering, and removal of nonbacterial 16S rRNA gene sequences, only samples with libraries of at least 100 quality-filtered sequences were analyzed. From the culture samples, two samples fell below this threshold and were removed from subsequent analyses (1 anoxic midintestine sample and 1 hypoxic lung sample). For the molecular samples, all vaginal, oral cavity, proximal intestine, and distal intestine sequence libraries met this criterion, but only five lung libraries remained (removed due to poor read quality profiles). The full data set included 176 biological samples, 15 blank extraction kit controls, and 17 negative swab controls, representing a total of 1,138 ASVs.

To confirm that the bacterial signals detected in the molecular surveys of mouse samples were legitimate, the composition and structure of the bacterial profiles of tissues and swabs were contrasted with those of blank (*n* = 15) and blank swab (*n* = 17) technical controls, using the adonis function in the *vegan* package. For each body site, the composition and structure of bacterial profiles were distinct from those of applicable negative controls (Table S11). Prominent ASVs (i.e., those with a ≥1% average relative abundance) in either blank or blank swab technical controls are shown in Fig. S7.

### Removal of background DNA contaminant ASVs with *decontam*.

After establishing that the 16S rRNA gene profiles of the tissue and swab samples from the pregnant mice were distinct from those of negative controls, the tissue and swab data sets were separately analyzed with *decontam* (117) to identify ASVs that were likely background DNA contaminants. Histogram plots of the distribution of prevalence scores indicated that a threshold of 0.8 would be appropriate for both data sets, thereby retaining a large percentage of ASVs (82% in the tissue data set and 72% in the swab data set). Between the two data sets, 209 ASVs were below the 0.8 threshold and identified as contaminants. Twenty-four ASVs were not detected in any biological samples from the molecular surveys, and 179 ASVs had an average relative abundance below 1% for each of the biological sample types. Three of the remaining six ASVs, *Ralstonia* (ASV 76), Streptococcus (ASV 520), and *Bacillus* (ASV 6), were detected as contaminants in the tissue and swab data sets. Streptococcus (ASV 11), *Muribacter* (ASV 4), and *Rodentibacter* (ASV 5), the three remaining ASVs, had average relative abundances above 1% in at least one body site from the opposing sample type (e.g., ASV 5 was above 1% average relative abundance in vaginal swabs but identified as a contaminant by *decontam* from the tissue data set), suggesting that they may be legitimate sequences, and they were not considered contaminants and were retained in subsequent analyses. To allow comparisons of the molecular data sets with the culture data sets, the *decontam* results were contrasted with the culture data to ensure that ASVs abundant in culture surveys were not removed as contaminants due to the fact that they were recovered via culture (i.e., they were legitimate as they were cultured by us). ASVs classified as contaminants through *decontam* were retained in subsequent analyses if they were either above 1% average relative abundance in at least one cultured body site or cultured from at least five mice for a given body site. Thirteen additional ASVs met these criteria, and ultimately, 16 of the 209 ASVs identified as potential contaminants by *decontam* were kept in the data sets for subsequent analyses. The 193 ASVs removed from the data set are listed in Table S12. One of the five lung samples dropped below the 100-sequence-read threshold following removal of contaminant ASVs and was removed from further analysis.

To aid comparisons of culture and molecular data sets, the molecular 16S rRNA gene profiles of the proximal and distal portions of the intestine were assessed for differences in their bacterial profiles, using the adonis function in *vegan*. No differences were observed for either composition (mouse identity, *P* = 0.19; intestine locale, *P* = 0.47) or structure (mouse identity, *P* = 0.14; intestine locale, *P* = 0.26), and these samples were bioinformatically pooled by mouse identity and considered “intestinal” samples from molecular surveys. These data were contrasted with those of cultures from the midintestine.

### Whole-genome sequencing and genomic analysis of isolates ASV 2 and ASV 5.

**(i) DNA extraction from bacterial isolates.** The 16S rRNA gene sequences associated with ASV 2 and ASV 5 were queried against a BLAST database of 16S rRNA gene sequences from isolates recovered and preserved during the previous study (21). Isolates with a 100% match were recovered from frozen stocks by plating 80 μL, using the medium and atmosphere they were originally recovered with (both isolates were recovered on chocolate agar plates and under hypoxic atmospheric conditions [5% O_2,_ 5% CO_2_, 90% N_2_]), and incubated for 48 h. Colonies were then collected using sterile inoculating loops into 500 μL of sterile PBS and centrifuged at 15,000 × *g* for 10 min. DNA extractions were performed using a DNeasy PowerLyzer PowerSoil kit (Qiagen, Valencia, CA, USA), and the first step included resuspension of the pelleted colonies in 500 μL of bead solution and adding the total resuspended volume to the bead tubes. Two additional modifications to the manufacturer’s protocol were made: (i) 100 μL of solutions C2 and C3 were combined into a single step followed by 5-min incubation at 4°C and subsequent centrifugation, and (ii) in the final elution step, DNA was eluted with 60 μL of solution C6 rather than 100 μL to increase DNA concentration. Extracted DNA was then stored at 4°C until submission (<48 h) for whole-genome sequencing (WGS).

### (ii) Construction and sequencing of sample DNA libraries.

Libraries were built by using the Illumina DNA Prep protocol and Nextera DNA CD indexes (Illumina). Libraries were sequenced at the Perinatology Research Branch, using iSeq 100 reagents (Illumina) with the iSeq 100 system (Illumina) and an output of 2 × 150-bp paired-end reads.

### (iii) DNA sequence processing and genome assembly.

Adapters from the raw sequence reads were removed by using Trimmomatic (v0.35). Genome assembly was performed using SPAdes (v 3.12.0) through the web-based Galaxy platform (118) with default parameters, except that k-mer sizes of 27, 37, 47, 57, 67, 77, and 87 were used. Contigs of less than 200 bp were removed after assembly, and the average coverage per contig was 429 for ASV 2 and 256 for ASV 5.

### (iv) Phylogenomic analysis of ASV 2 and ASV 5 isolates in the context of other *Rodentibacter* genomes.

Phylogenomic analysis was performed by using the up-to-date bacterial core gene (UBCG) tool (119) for phylogenomic tree inference, which utilized 92 core genes to assess phylogenomic relationships. The assembled genomes for isolates of ASV 2 and ASV 5, along with published *Rodentibacter* type strain genomes (downloaded through NCBI GenBank’s ftp server), and secondarily with all published *Rodentibacter* genomes (at the time of analysis), were processed through the UBCG pipeline by using default parameters in VirtualBox. For the phylogenomic tree featuring only published type strains, Muribaculum intestinale was included as an outgroup and Muribacter muris was included as a within-family (*Pasteurellaceae*) outgroup. Trees were rooted on the midpoint, using FigTree (v1.4.4) (120).

### (v) Genome annotation and analysis of ASV 2 and ASV 5 isolates.

Prior to genome annotation, the contigs for each assembled genome were reordered and aligned to their closest strain, *R. pneumotropicus* strain P441 and *R. heylii* strain G1 for isolates ASV 2 and ASV 5, respectively, using Mauve multiple-genome-alignment software (v 20150226) (121). The 16 published genomes of *R. pneumotropicus* along with isolate ASV 2 were annotated with Prokka (v 1.14.5) (77) and processed through Roary (v 3.13.0) (122) to establish a core genome for *R. pneumotropicus* and to subsequently assess the representation of this core genome in the genome of isolate ASV 2. This process was repeated for isolate ASV 5 and the seven published genomes of *R. heylii*. Prokka and Roary tools were run through Galaxy with default parameters except that paralog genes were not split when Roary was used. For functional and pathway analysis, the genomes of isolates ASV 2 and ASV 5 were annotated with NCBI’s Prokaryotic Genome Annotation Pipeline (PGAP) (123) tool using default parameters. These annotated genomes were submitted to the KEGG Automatic Annotation Server (KAAS) (124) for KEGG pathway enrichment analysis, using only bacteria included in the representative set of “Prokaryotes” as the template data set for KO assignment and a bidirectional best-hit assignment method. For detailed metabolic comparisons of complete pathways, pathway/genome databases (PGDBs) were generated using the assembled genomes for isolates ASV 2 and ASV 5 with Pathway Tools software (v 25.0) (125). Default parameters were used; however, several manual refinement steps were required per the user’s guide, including assigning probable enzymes and modified proteins, predicting transcription units, and inferring probable transporters.

### Statistical analysis.

**(i) Alpha diversity.** The alpha diversities of bacterial culture profiles for each body site under each atmosphere were characterized by the Chao1 (richness), Shannon (evenness), and inverse Simpson (evenness) indices, and variation in diversity was assessed for culture profiles under each atmosphere separately for each body site. Then, after mice that had culture profiles from three atmospheres for all four body sites were bioinformatically pooled (*n* = 5), diversity was compared between body sites. When all three atmosphere culture profiles were compared, data sets for each body site were rarefied to their lowest read depths (oral cavity, 3,976 reads; lung, 358 reads; intestine, 4,745 reads; vagina, 7,719 reads). When diversity between body sites was compared, each individual mouse’s pooled samples were rarefied to the lowest read depth (20,954 reads). Alpha diversities of sequenced microbiota (molecular profiles) of the oral cavity, intestine, and vagina were also compared in the same way and rarefied to the lowest read depth (810 reads); the lung was excluded from this analysis due to low sample size (*n* = 4). Rarefaction was performed in R with the *phyloseq* package. Alpha diversities were calculated and visualized in R using the *phyloseq* package and labeled in Adobe Illustrator. Alpha diversities were statistically evaluated with the *rstatix* package in R by repeated-measures analysis of variance (ANOVA) or Friedman’s ANOVA followed by paired *t* tests or Wilcoxon signed-rank tests when appropriate.

### (ii) Beta diversity.

Statistical comparisons of beta diversities were performed using the *vegan* and *pairwiseAdonis2* packages in RStudio (v 1.3.1093) and R (v. 4.0.3). Nonparametric multivariate analysis of variance (NPMANOVA) was used to evaluate the composition and structure of bacterial profiles by using the Jaccard and Bray-Curtis dissimilarity indices, respectively. For comparisons where variation by mouse identity was observed, it was secondarily controlled for using the “strata” term on mouse identity in the adonis and pairwise.adonis2 functions in the *vegan* and *pairwiseAdonis2* packages, respectively. The composition and structure of bacterial profiles were visualized with principal-coordinate analysis (PCoA) plots generated using the RAM package in R (v. 4.0.3).

### (iii) LEfSe.

LEfSe analyses were performed to identify features, taxa (assessed as hierarchical analyses), or ASVs (assessed as ASV-only analyses) that were preferentially recovered in different atmospheres for each body site and secondarily in each body site after bioinformatically pooling culture data from each atmosphere. To identify taxa that were differentially abundant in the hierarchical analysis, each taxonomic level from phylum to species was included for each individual ASV, when available. In assessments of bacterial profile features preferentially recovered in one atmosphere over the other two, or in one body site over the other three, only mice with cultures in all three atmospheres from a body site were included (*n* = 9 for oral cavity, intestine, and vagina; *n* = 7 for lung).

For all LEfSe analyses, singleton features were removed from each data set, multiclass analysis of all-against-all was used only in identifying features that were preferentially abundant in one condition over all the others, and only features with an LDA score above 3.0 were considered preferentially abundant. Histograms (ASV-only analyses) and cladograms (hierarchical analyses) were generated by using the Galaxy hub. Each taxon is indicated on cladograms when identified as a significant feature except order (to avoid visual congestion).

### (iv) Mantel tests.

Mantel tests were used to determine whether there was a correlation between the structure of bacterial culture profiles and the structure of molecular profiles for each body site. Only mice with bacterial profiles in both culture and molecular data sets in a body site were evaluated. Mantel tests were performed on Bray-Curtis distance matrices, using the *vegan* package in RStudio (v 1.3.1093) and R (v. 4.0.3).

### (v) Figures.

Heatmaps were generated using gplots and Heatplus packages in R (v. 4.0.3), and clustering of samples was performed on Bray-Curtis dissimilarity distance matrices, using an unweighted pair group method with arithmetic mean (UPGMA) in the hclust function in R.

### Data availability.

Original sample-specific MiSeq run files are available in the Short Read Archive from the original study by Theis et al. (21) (BioProject identifier [ID] PRJNA594727). Raw sequence files and the assembled genomes of isolates ASV 2 and ASV 5 with annotations from NCBI’s Prokaryotic Genome Annotation Pipeline are available at BioProject ID PRJNA823350 under BioSamples SAMN27293572 and SAMN27294279, respectively.
