# Supplementary material for: Microbiota of the Pregnant Mouse: Characterization of the Bacterial Communities in the Oral Cavity, Lung, Intestine, and Vagina through Culture and DNA Sequencing
Source: Microbiol Spectr. 2022 Aug 2;10(4):e01286-22. doi: 10.1128/spectrum.01286-22 (PMC9430855; doi:10.1128/spectrum.01286-22)
Supplement: Supplemental file 1 — Supplemental material. Download spectrum.01286-22-s0001.pdf, PDF file, 3.5 MB [file spectrum.01286-22-s0001.pdf]

Microbiota of the pregnant mouse: characterization of the bacterial communities in the oral cavity, lung, intestine, and vagina through culture and DNA sequencing

RUNNING TITLE: Pregnant mouse microbiota

Jonathan M. Greenberg[1,2], Roberto Romero[1,3-6], Andrew D. Winters[1,7,8], Jose Galaz[1,2], Valeria Garcia-Flores[1,2], Marcia Arenas-Hernandez[1,2], Jonathan Panzer[1,7], Zachary Shaffer[1,8], David J. Kracht[1,8], Nardhy Gomez-Lopez[1,2,7*], Kevin R. Theis[1,2,7*]

AUTHOR AFFILIATIONS

[1] Perinatology Research Branch, Division of Obstetrics and Maternal-Fetal Medicine, Division of Intramural Research, *Eunice Kennedy Shriver* National Institute of Child Health and Human Development, National Institutes of Health, U.S. Department of Health and Human Services, Bethesda, MD, and Detroit, MI, United States

[2] Department of Obstetrics and Gynecology, Wayne State University School of Medicine, Detroit, MI, United States

[3] Department of Obstetrics and Gynecology, University of Michigan, Ann Arbor, MI, United States

[4] Department of Epidemiology and Biostatistics, Michigan State University, East Lansing, MI, United States

[5] Center for Molecular Medicine and Genetics, Wayne State University, Detroit, MI, United States

[6] Detroit Medical Center, Detroit, MI, United States

[7] Department of Biochemistry, Microbiology, and Immunology, Wayne State University School of Medicine, Detroit, MI, United States

[8] Department of Physiology, Wayne State University School of Medicine, Detroit, MI, United States

CORRESPONDING AUTHOR(S)

*** Kevin R. Theis, ktheis@med.wayne.edu**

*** Nardhy Gomez-Lopez, ngomezlo@med.wayne.edu**

Supplemental Table 1. Description of previous 16S rRNA gene studies of the murine oral microbiota.

Author(s)	Year	Culture methods	Mouse strain	Mouse sex	Key microbiota findings
Abusleme, et al. (1)	2017	Not performed	C57BL/6	Not indicated	The most abundant genera observed in the oral cavity were *Lactobacillus*, *Staphyloccocus*, *Enterococcus*, *Rothia*, *Acinetobacter*, and *Streptococcus*.
Hernández-Arriaga, et al. (2)	2019	Not performed	C57BL/6	Exclusively male	Relatively abundant genera varied with age and sampling method. Relatively abundant genera included *Actinobacillus*, *Actinomyces*, *Neisseria*, *Corynebacterium*, *Aggregatibacter*, *Staphylococcus*, *Streptococcus*, and *Propionibacterium. Rodentibacter* was observed in some mice.
Abusleme, et al. (3)	2020	Not performed	C57BL/6	Not indicated	Bacteria relatively abundant in adult mice included *Streptococcus*, *Lactobacillus* spp., *Turicibacter*, *Staphylococcus*, *Enterococcus*, and *Flavobacterium.* Bacterial composition was distinct for mice from different laboratories.
Jašarević, et al. (4)	2021	Not performed	C57BL/6	Exclusively female	Prominent taxa were Lachnospiraceae, Bacteroidales, *Oscillibacter*, and *Draconibacterium.*

Supplemental Table 2. Description of previous 16S rRNA gene studies of the murine lung microbiota.

Author(s)	Year	Culture methods	Mouse strain	Mouse sex	Key microbiota findings
Barfod, et al. (5)	2013	200uL of bronchoalveolar lavage (BAL) fluid was cultured on general growth media blood agar 5% and Chocolate agar at 37ºC for 24 hours and under hypoxic conditions (5% CO_2, 3% H_2, 5% O_2 and 87% N_2) for 48 hours and identified by a Vitek2 system	BALB/cJ	Exclusively female	12 core genera were observed between lung, cecum, and vaginal samples. The lung and vagina shared many genera. *Staphylococcus*, *Massilia*, *Corynebacterium*, *Pseudomonas*, *Streptococcus*, and *Sphingomonas* were detected in all lung samples. Only *Micrococcus luteus* was recovered via culture.
Poroyko, et al. (6)	2015	A single drop of BAL fluid was added to chopped meat carbohydrate broth, PR II, tryptic soy agar with 5% sheep blood, and chocolate II agar plates and incubated at 5% CO_2 at 37ºC and Brucella agar with 5% sheep blood, hemin, and vitamin K1 incubated under anaerobic conditions. Plates were checked daily for 4 days and broth was checked daily for 14 days.	C57BL/6	Not indicated	Molecular surveys detected a high relative abundance of the family Alicyclobacillaceae. *Stenotrophomonas* and *Ochrobactrum* were observed in culture and molecular surveys.
Singh, et al. (7)	2017	Not performed	C57BL/6N	Not indicated	Abundant genera observed in adult mice included *Actinobacillus*, *Achromobacter*, *Lactobacillus*, *Bacillus*, and *Streptococcus*.
Dickson, et al. (8)	2018	Not performed	C57BL/6	Exclusively female	No core lung microbiome was observed. Environmental factors such as cohabitation and supplying vendor were primary drivers in microbiome composition. Most abundant taxa observed include

					Pseudomonas, *Lactobacillus*, *Streptococcus* and *Turicibacter*.
Kostric, et al. (9)	2018	Not performed	BALB/c	Exclusively female	Abundant genera in adult (2-9 mo) mice included *Rhodococcus*, *Staphylococcus*, *Lactobacillus*, *Streptococcus*, *Delftia*, *Propionibacterium*, *Pseudomonas*, *Diaphorobacter*, *Defluvibacter*, *Ochrobactrum*, *Mucispirillium*.
Ashley, et al. (10)	2020	Not performed	C57BL/6	Exclusively female	Abundant genera observed in control mice included *Pseudomonas, Lactobacillus,* and *Bacteroides.*
Li, et al. (11)	2020	Not performed	C57BL/6N	Exclusively male	Microbiota characterization was limited to phyla.
Baker, et al. (12)	2021	Not performed	C57BL/6	Exclusively female	Top taxa were *Turicibacter, Lactobacillus, Flavobacterium*, and *Alistipes*.
Lipinski, et al. (13)	2021	Not performed	C57BL/6	Exclusively female	*Sphingomonas* and *Burkholderia* were the most relatively abundant taxa among control mice.

Supplemental Table 3. Description of previous 16S rRNA gene studies of the murine vaginal microbiota.

Author(s)	Year	Culture methods	Mouse strain	Mouse sex	Key microbiota findings
Barfod, et al. (5)	2013	Not performed	BALB/cJ	N/A	Vaginal samples from mice clustered into two groups and were typically abundant in *Streptococcus.* Other notable genera included *Acinetobacter, Sphingomonas, Enterococcus*, and *Polaromonas*.
Oh, et al. (14)	2016	Vaginal wash fluids were spread onto CNA and MacConkey agar plates at 35ºC for 48h under oxic conditions and on anaerobic basal agar Oxoid plates at 37ºC for 5 days under anaerobic conditions.	C57BL/6	N/A	Vaginal washes from control mice were composed of AY538685, *Proteus, Escherichia, Enterobacter, Serratia,* and *Staphylococcus*. Culture was used strictly to assess bacterial load.
Vrbanac, et al. (15)	2018	Not performed	C57BL/6J	N/A	Five distinctive community states of the vaginal microbiota were described. These community states were defined by abundance in *Staphylococcus* and/or *Enterococcus*, *Lactobacillus*, or a mixed population. The stage of estrus did not impact vaginal community structure.
Karpinets, et al. (16)	2020	Not performed	C57BL/6	N/A	Relatively abundant genera found in control mice included *Bacteroides, Staphylococcus, Turicibacter, Streptococcus, Paraliobacillus, Lachnoclostridium, Paeniclostridium, Halorubellus, Halorubrum, Lactobacillus, Oscillibacter, Romboutsia, Parabacteroides, Atopostipes, Akkermansia, and Sporosarcina.*
Hernandez-Quiroz, et al. (17)	2021	Not performed	FVB	N/A	Relatively abundant taxa included *Streptococcus, Microbacterium, Kaistobacter, Aggregatibacter*, and Weeksellaceae.

Jašarević, et al. (4)	2021	Not performed	C57BL/6	N/A	The vagina was most relatively abundant in *Pasteurella pneumotropica* (recently reclassified as *Rodentibacter*).
Mejia, et al. (18)	2022	Not performed	C57BL/6J	N/A	Similar to Vrbanac, et al., microbiota were classified into CSTs according to the predominant taxa *Staphylococcus*, *Enterococcus*, or *Lactobacillus.*

Supplemental Table 4. Comparisons of the bacterial profiles of cultured murine sample microbiota under anoxic, hypoxic, and oxic conditions.

**Beta diversity**	**Composition**			**Structure**		
	**F**	**R^2**	**p**	**F**	**R^2**	**p**
**Anoxic**						
Mouse ID	1.049	0.224	0.299	1.107	0.207	0.252
Body site	3.745	0.240	**0.001**	5.830	0.327	**0.001**
**Hypoxic**						
Mouse ID	1.190	0.268	**0.019**	0.766	0.195	0.923
Body site	2.845	0.192	**0.001**	2.548	0.194	**0.001**
**Oxic**						
Mouse ID	1.089	0.249	0.175	1.307	0.309	0.101
Body site	2.936	0.202	**0.001**	1.764	0.125	**0.039**

Supplemental Table 5. Comparisons of the (A) composition and (B) structure of the cultured microbiota between different atmospheres for the oral cavity, lung, intestine, and vagina.

**A**	**Composition**											
**Bacterial composition**	**All atmospheres**			**Oxic x Hypoxic**			**Oxic x Anoxic**			**Hypoxic x Anoxic**		
	**F**	**R^2**	**p**	**F**	**R^2**	**p**	**F**	**R^2**	**p**	**F**	**R^2**	**p**
**Oral cavity**												
Mouse ID	0.981	0.327	0.569	1.201	0.535	0.089	0.784	0.448	0.970	0.980	0.481	0.544
Atmosphere	1.581	0.106	**0.015**	1.387	0.069	0.111	1.659	0.095	**0.040**	1.583	0.078	**0.042**
**Lung**												
Mouse ID	1.039	0.348	0.316	0.904	0.507	0.841	1.050	0.515	0.307	1.147	0.565	0.131
Atmosphere	1.268	0.094	0.070									
**Intestine**												
Mouse ID	1.029	0.280	0.381	0.974	0.463	0.570	1.016	0.412	0.437	1.127	0.449	0.205
Atmosphere	3.435	0.207	**0.001**	1.150	0.061	0.264	5.038	0.227	**0.001**	4.456	0.197	**0.001**
**Vagina**												
Mouse ID[†]	1.911	0.511	**0.001**									
Atmosphere[‡]	1.142	0.061	0.202	0.682	0.039	0.566	1.059	0.056	0.084	0.938	0.052	0.063

**B**	**Structure**											
**Bacterial structure**	**All atmospheres**			**Oxic x Hypoxic**			**Oxic x Anoxic**			**Hypoxic x Anoxic**		
	**F**	**R^2**	**p**	**F**	**R^2**	**p**	**F**	**R^2**	**p**	**F**	**R^2**	**p**
**Oral cavity**												
Mouse ID	1.005	0.296	0.494	2.794	0.719	**0.013***	0.706	0.359	0.861	0.617	0.343	0.956
Atmosphere	3.434	0.202	**0.007**	1.832	0.052	0.138	4.597	0.234	**0.004**	2.836	0.157	**0.026**
**Lung**												
Mouse ID	0.847	0.302	0.774	0.801	0.481	0.811	0.931	0.490	0.621	0.886	0.502	0.708
Atmosphere	1.313	0.104	0.167									
**Intestine**												
Mouse ID	1.205	0.300	0.202	1.058	0.488	0.363	1.072	0.384	0.371	1.537	0.506	0.097
Atmosphere	4.161	0.230	**0.001**	0.969	0.050	0.425	7.464	0.297	**0.001**	5.528	0.202	**0.001**
**Vagina**												
Mouse ID[†]	22.71	0.917	**0.001**									
Atmosphere[⁑]	2.271	0.018	0.053	0.188	0.011	0.063	0.587	0.032	**0.004**	0.265	0.015	**0.023**

[†]Mouse identity was controlled for in atmosphere pairwise comparisons.

[‡]Atmosphere was not significant globally for bacterial composition in the vagina when controlling for mouse identity in a subsequent analysis: F = 0.898, R^2 = 0.065, p = 0.110.

[⁑]Atmosphere was significant globally for bacterial structure in the vagina when controlling for mouse identity in a subsequent analysis: F = 0.350, R^2 = 0.026, p = **0.001**.

*Oral cavity: Oxic vs hypoxic conditions were not significant when controlling for mouse identity in a subsequent analysis: F = 0.681 R^2 = 0.039 p = 0.303.

Supplemental Table 6. Comparisons of the cultured murine microbiota under (A) anoxic, (B) hypoxic, and (C) oxic conditions.

**A**		Composition											
Anoxic atmosphere		**Oral cavity n = 11**			**Lung n = 9**			**Intestine n = 9**			**Vagina n = 10**		
		**F**	**R^2**	**p**	**F**	**R^2**	**p**	**F**	**R^2**	**p**	**F**	**R^2**	**p**
**Oral cavity**	Mouse ID				1.07	0.52	0.281	1.07	0.39	0.386	0.94	0.44	0.724
	Body site				2.08	0.10	**0.004**	8.46	0.31	**0.001**	2.83	0.13	**0.001**
Structure: **Lung**	Mouse ID	1.14	0.51	0.281				1.06	0.46	0.358	1.05	0.56	0.264
	Body site	3.04	0.14	**0.006**				3.94	0.19	**0.001**	1.42	0.08	**0.041**
Structure: **Intestine**	Mouse ID	1.00	0.34	0.485	1.06	0.43	0.431				1.13	0.51	0.18
	Body site	10.98	0.38	**0.001**	5.71	0.26	**0.004**				3.95	0.18	**0.002**
Structure: **Vagina**	Mouse ID	0.90	0.40	0.588	1.28	0.55	0.208	1.14	0.46	0.326			
	Body site	4.42	0.20	**0.005**	3.46	0.15	**0.012**	6.54	0.26	**0.002**			

**B**		Composition*											
Hypoxic atmosphere		**Oral cavity n = 10**			**Lung n = 9**			**Intestine n = 10**			**Vagina n = 9**		
		**F**	**R^2**	**p**	**F**	**R^2**	**p**	**F**	**R^2**	**p**	**F**	**R^2**	**p**
**Oral cavity**	Mouse ID												
	Body site				2.01	0.11	**0.004**	4.54	0.20	**0.002**	2.99	0.15	**0.004**
Structure: **Lung**	Mouse ID	1.10	0.52	0.288									
	Body site	3.02	0.14	**0.012**				2.69	0.14	**0.004**	1.19	0.07	0.188
Structure: **Intestine**	Mouse ID	0.68	0.343	0.928	0.82	0.47	0.841						
	Body site	2.64	0.15	**0.049**	2.08	0.12	**0.038**				3.37	0.17	**0.002**
Structure: **Vagina**	Mouse ID	0.78	0.42	0.811	0.89	0.50	0.654	1.09	0.47	0.343			
	Body site	1.51	0.09	0.185	2.96	0.17	**0.025**	2.88	0.14	**0.019**			

**C**		Composition											
Oxic atmosphere		**Oral cavity n = 9**			**Lung n = 9**			**Intestine n = 10**			**Vagina n = 10**		
		**F**	**R^2**	**p**	**F**	**R^2**	**p**	**F**	**R^2**	**p**	**F**	**R^2**	**p**
**Oral cavity**	Mouse ID				1.09	0.51	0.243	1.00	0.42	0.490	0.99	0.44	0.498
	Body site				2.44	0.13	**0.001**	4.22	0.20	**0.001**	3.32	0.16	**0.001**
Structure: **Lung**	Mouse ID	1.23	0.57	0.252				1.17	0.54	0.090	1.18	0.58	0.072
	Body site	1.40	0.07	0.213				2.87	0.13	**0.001**	1.60	0.08	**0.021**
Structure: **Intestine**	Mouse ID	0.81	0.43	0.746	1.46	0.62	**0.032**†				0.99	0.43	0.537
	Body site	1.56	0.09	0.161	1.95	0.08	**0.030**				2.99	0.14	**0.001**
Structure: **Vagina**	Mouse ID	0.91	0.46	0.591	2.19	0.73	**0.023**‡	0.93	0.42	0.577			
	Body site	1.53	0.09	0.218	1.20	0.04	0.323	2.51	0.13	**0.042**			

*Because mouse identity had a significant effect on composition under hypoxic conditions, in panel B, all pairwise comparisons of microbiota composition were controlled for mouse identity using the strata function in adonis.

†Lung vs intestine under oxic atmosphere: Body site was significant when controlling for mouse identity in a subsequent analysis: F = 1.769, R^2 = 0.094, p = **0.039**.

‡Vagina vs lung under oxic atmosphere: Body site was non-significant when controlling for mouse identity in a subsequent analysis: F = 0.817, R^2 = 0.046, p = 0.426.

Supplemental Table 7. Prominent amplicon sequence variants (ASVs) shared among cultures from the oral cavity, lung, intestine, and vagina.

Taxonomic classification	ASV	Body site with greatest average relative abundance	Atmosphere with greatest average relative abundance among all body sites
Rodentibacter	ASV 2	Vagina (39.4%)	Oxic (41.4%)
Lactobacillus	ASV 3	Lung (26.8%)	Anoxic (20.0%)
Rodentibacter	ASV 5	Vagina (31.4%)	Oxic (17.9%)
Staphylococcus	ASV 17	Oral cavity (10.4%)	Hypoxic (9.0%)
Rothia nasimurium	ASV 79	Vagina (0.93%)	Oxic (1.1%)

Supplemental Table 8. Amplicon sequence variants (ASVs) that were prominent in cultures from only a single body site.

Taxonomic classification	ASV	Body site	Atmosphere with greatest average relative abundance
Streptococcus danieliae	ASV 1	Lung	Anoxic (1.2%)
Staphylococcus	ASV 8	Vagina	Hypoxic (5.7%)
Escherichia / Shigella	ASV 10	Lung	Anoxic (13.6%)
Streptococcus	ASV 11	Oral	Anoxic (2.4%)
Parasutterella	ASV 20	Intestine	Anoxic (2.2%)
Bifidobacterium	ASV 41	Intestine	Anoxic (1.8%)
Bacteroides acidifaciens	ASV 53	Intestine	Anoxic (3.0%)
Acinetobacter	ASV 60	Intestine	Oxic (7.1%)
Pseudomonas	ASV 73	Intestine	Oxic (8.4%)
Actinobacteria	ASV 87	Lung	Oxic / Hypoxic (6.2%)
Streptomycetaceae	ASV 97	Lung	Oxic (9.9%)
Actinobacteria	ASV 106	Lung	Oxic / Hypoxic (4.8%)
Brevibacillus	ASV 108	Lung	Hypoxic (7.1%)
Streptococcus	ASV 116	Vagina	Anoxic (1.2%)
Bacillus	ASV 123	Intestine	Oxic (4.0%)
Enterorhabdus	ASV 125	Lung	Anoxic (6.0%)
Muribacter muris	ASV 143	Oral	Oxic (1.1%)
Brevibacillus limnophilus	ASV 156	Lung	Hypoxic (3.8%)
Coriobacteriaceae UCG-002	ASV 180	Intestine	Anoxic (1.1%)
Paenibacillus	ASV 361	Intestine	Oxic (1.2%)

Supplemental Table 9. Amplicon sequence variant (ASV) counts from culture and molecular datasets.

	**Culture**	**Molecular**	**Shared**	**% Shared**
**Oral cavity**	177	454	122	24%
**Lung**	152	93	44	22%
**Intestine**	331	660	262	36%
**Vagina**	134	510	93	17%
**All body sites**	411	751	339	41%

Supplemental Table 10. Summary table of the genomes of the two *Rodentibacter* isolates identified in the study alongside type strains and their closest relatives.

	Rodentibacter pneumotropicus			***Rodentibacter heylii***		
	Type strain	Closest relative	Our strain	Type strain	Closest relative	Our strain
**ID**	ATCC 35149	P441	ASV2	ATCC 12555	G1	ASV5
**Genome (Mbp)**	2.43	2.4	2.45	2.72	2.61	2.51
**G+C content (%)**	39.87	39.81	39.9	40.2	40.19	40.13
**Total genes**	2,297	2,296	2,384	2,656	2,520	2,474
**Percent of core genome represented**	100%	100%	100%	100%	100%	99.7% (1644 / 1649 genes)
**Hypothetical proteins**	261	286	308	360	370	285

Supplemental Table 11. Comparisons of the (A) swab and (B) tissue sample cultured microbiota to technical controls.

A				
**Negative swab extraction controls**	**Oral cavity**		**Vagina**	
	**F**	**p**	**F**	**p**
**Composition**	3.569	**0.001**	2.482	**0.001**
**Structure**	17.046	**0.001**	8.344	**0.001**

B						
**Negative extraction controls**	**Lung**		**Proximal Intestine**		**Distal Intestine**	
	**F**	**p**	**F**	**p**	**F**	**p**
**Composition**	1.521	**0.018**	9.723	**0.001**	10.021	**0.001**
**Structure**	2.414	**0.002**	7.957	**0.001**	8.955	**0.001**

Supplemental Table 12. Amplicon sequence variants (ASVs) identified as likely background DNA contaminants in the tissue and swab sample datasets.

Identified as contaminant

Tissue.control	Swab.control	Both	ASV
TRUE	TRUE	TRUE	ASV6.Bacteria.Firmicutes.Bacilli.Bacillales.Bacillaceae.Bacillus.NA
TRUE	TRUE	TRUE	ASV9.Bacteria.Firmicutes.Bacilli.Lactobacillales.Lactobacillaceae.Lactobacillus.NA
FALSE	TRUE	FALSE	ASV14.Bacteria.Proteobacteria.Gammaproteobacteria.Pseudomonadales.Pseudomonadaceae.Pseudomonas.NA
TRUE	TRUE	TRUE	ASV38.Bacteria.Proteobacteria.Gammaproteobacteria.Enterobacterales.Enterobacteriaceae.Enterobacteriaceae Unclassified.NA
FALSE	TRUE	FALSE	ASV69.Bacteria.Bacteroidota.Bacteroidia.Bacteroidales.Muribaculaceae.Muribaculaceae Unclassified.NA
FALSE	TRUE	FALSE	ASV70.Bacteria.Firmicutes.Clostridia.Lachnospirales.Lachnospiraceae.Lachnoclostridium.NA
FALSE	TRUE	FALSE	ASV75.Bacteria.Proteobacteria.Gammaproteobacteria.Pseudomonadales.Pseudomonadaceae.Pseudomonas.NA
TRUE	TRUE	TRUE	ASV76.Bacteria.Proteobacteria.Gammaproteobacteria.Burkholderiales.Burkholderiaceae.Ralstonia.NA
FALSE	TRUE	FALSE	ASV90.Bacteria.Firmicutes.Clostridia.Lachnospirales.Lachnospiraceae.Lachnospiraceae Unclassified.NA
FALSE	TRUE	FALSE	ASV101.Bacteria.Firmicutes.Clostridia.Lachnospirales.Lachnospiraceae.Lachnospiraceae FCS020 group.NA
TRUE	FALSE	FALSE	ASV122.Bacteria.Actinobacteriota.Coriobacteriia.Coriobacteriales.Eggerthellaceae.Enterorhabdus.NA
FALSE	TRUE	FALSE	ASV128.Bacteria.Firmicutes.Clostridia.Lachnospirales.Lachnospiraceae.Lachnospiraceae NK4A136 group.bacterium
FALSE	TRUE	FALSE	ASV153.Bacteria.Firmicutes.Clostridia.Lachnospirales.Lachnospiraceae.Lachnospiraceae Unclassified.NA
TRUE	FALSE	FALSE	ASV160.Bacteria.Firmicutes.Clostridia.Peptostreptococcales-Tissierellales.Peptostreptococcaceae.Romboutsia.ilealis
FALSE	TRUE	FALSE	ASV162.Bacteria.Firmicutes.Bacilli.Lactobacillales.Lactobacillaceae.Lactobacillus.NA
TRUE	FALSE	FALSE	ASV168.Bacteria.Firmicutes.Bacilli.Lactobacillales.Lactobacillaceae.Lactobacillus.NA
FALSE	TRUE	FALSE	ASV191.Bacteria.Firmicutes.Clostridia.Clostridia vadinBB60 group.NA.NA Unclassified.NA
FALSE	TRUE	FALSE	ASV197.Bacteria.Firmicutes.Clostridia.Lachnospirales.Lachnospiraceae.Lachnospiraceae Unclassified.NA

TRUE	FALSE	FALSE	ASV207.Bacteria.Proteobacteria.Gammaproteobacteria.Pseudomonadales.Pseudomonadaceae.Pseudomonas.NA
FALSE	TRUE	FALSE	ASV210.Bacteria.Firmicutes.Clostridia.Clostridia vadinBB60 group.NA.NA Unclassified.NA
FALSE	TRUE	FALSE	ASV224.Bacteria.Firmicutes.Clostridia.Lachnospirales.Lachnospiraceae.Lachnospiraceae Unclassified.NA
FALSE	TRUE	FALSE	ASV225.Bacteria.Firmicutes.Clostridia.Lachnospirales.Lachnospiraceae.Roseburia.NA
TRUE	TRUE	TRUE	ASV229.Bacteria.Proteobacteria.Gammaproteobacteria.Pseudomonadales.Pseudomonadaceae.Pseudomonas.NA
FALSE	TRUE	FALSE	ASV234.Bacteria.Firmicutes.Clostridia.Oscillospirales.Ruminococcaceae.[Eubacterium] siraeum group.NA
TRUE	TRUE	TRUE	ASV238.Bacteria.Proteobacteria.Gammaproteobacteria.Burkholderiales.Comamonadaceae.Comamonadaceae Unclassified.NA
FALSE	TRUE	FALSE	ASV240.Bacteria.Firmicutes.Clostridia.Oscillospirales.NA.NA Unclassified.NA
FALSE	TRUE	FALSE	ASV242.Bacteria.Firmicutes.Clostridia.Lachnospirales.Lachnospiraceae.Lachnospiraceae NK4A136 group.NA
FALSE	TRUE	FALSE	ASV250.Bacteria.Firmicutes.Clostridia.Oscillospirales.Ruminococcaceae.Incertae Sedis.NA
FALSE	TRUE	FALSE	ASV264.Bacteria.Firmicutes.Bacilli.Bacillales.NA.NA Unclassified.NA
TRUE	TRUE	TRUE	ASV267.Bacteria.Firmicutes.Bacilli.Bacillales.Bacillaceae.Bacillaceae Unclassified.NA
FALSE	TRUE	FALSE	ASV269.Bacteria.Firmicutes.Clostridia.Lachnospirales.Lachnospiraceae.Lachnoclostridium.NA
FALSE	TRUE	FALSE	ASV273.Bacteria.Firmicutes.Clostridia.Lachnospirales.Lachnospiraceae.Lachnospiraceae Unclassified.NA
TRUE	TRUE	TRUE	ASV274.Bacteria.Proteobacteria.Alphaproteobacteria.Rhizobiales.Xanthobacteraceae.Xanthobacteraceae Unclassified.NA
TRUE	FALSE	FALSE	ASV281.Bacteria.Proteobacteria.Gammaproteobacteria.Enterobacterales.Enterobacteriaceae.Enterobacteriaceae Unclassified.NA
FALSE	TRUE	FALSE	ASV284.Bacteria.Firmicutes.Clostridia.Oscillospirales.Ruminococcaceae.Ruminococcaceae Unclassified.NA
FALSE	TRUE	FALSE	ASV309.Bacteria.Firmicutes.Clostridia.Oscillospirales.Oscillospiraceae.Colidextribacter.NA
FALSE	TRUE	FALSE	ASV312.Bacteria.Firmicutes.Bacilli.Bacillales.NA.NA Unclassified.NA

FALSE	TRUE	FALSE	ASV321.Bacteria.Firmicutes.Clostridia.Oscillospirales.Butyricicoccaceae.Butyricicoccus.NA
TRUE	TRUE	TRUE	ASV322.Bacteria.Proteobacteria.Gammaproteobacteria.Xanthomonadales.Xanthomonadaceae.Stenotrophomonas.NA
TRUE	TRUE	TRUE	ASV338.Bacteria.Proteobacteria.Gammaproteobacteria.Pseudomonadales.Pseudomonadaceae.Pseudomonas.NA
TRUE	FALSE	FALSE	ASV347.Bacteria.Firmicutes.Clostridia.Lachnospirales.Lachnospiraceae.Lachnospiraceae Unclassified.NA
TRUE	FALSE	FALSE	ASV350.Bacteria.Firmicutes.Clostridia.Peptostreptococcales-Tissierellales.Peptostreptococcaceae.Romboutsia.NA
FALSE	TRUE	FALSE	ASV353.Bacteria.Firmicutes.Clostridia.Clostridia vadinBB60 group.NA.NA Unclassified.NA
FALSE	TRUE	FALSE	ASV354.Bacteria.Firmicutes.Clostridia.Lachnospirales.Lachnospiraceae.A2.NA
FALSE	TRUE	FALSE	ASV356.Bacteria.Firmicutes.Clostridia.Lachnospirales.Lachnospiraceae.Lachnospiraceae Unclassified.NA
TRUE	FALSE	FALSE	ASV359.Bacteria.Firmicutes.Clostridia.Lachnospirales.Lachnospiraceae.Lachnospiraceae Unclassified.NA
FALSE	TRUE	FALSE	ASV360.Bacteria.Firmicutes.Bacilli.Bacillales.Bacillaceae.Pseudogracilibacillus.auburnensis
TRUE	TRUE	TRUE	ASV363.Bacteria.Actinobacteriota.Actinobacteria.Micrococcales.Microbacteriaceae.Leifsonia.NA
FALSE	TRUE	FALSE	ASV370.Bacteria.Firmicutes.Clostridia.Oscillospirales.Oscillospiraceae.Oscillospiraceae Unclassified.NA
FALSE	TRUE	FALSE	ASV382.Bacteria.Firmicutes.Symbiobacteriia.Symbiobacteriales.NA.NA Unclassified.NA
TRUE	TRUE	TRUE	ASV391.Bacteria.Proteobacteria.Gammaproteobacteria.Burkholderiales.Comamonadaceae.Comamonadaceae Unclassified.NA
TRUE	FALSE	FALSE	ASV397.Bacteria.Firmicutes.Clostridia.Lachnospirales.Lachnospiraceae.Roseburia.NA
TRUE	FALSE	FALSE	ASV406.Bacteria.Firmicutes.Clostridia.Clostridia UCG-014.NA.NA Unclassified.NA
FALSE	TRUE	FALSE	ASV413.Bacteria.Firmicutes.Clostridia.Clostridia UCG-014.NA.NA Unclassified.NA
TRUE	FALSE	FALSE	ASV434.Bacteria.Firmicutes.Bacilli.Lactobacillales.Leuconostocaceae.Weissella.NA
TRUE	TRUE	TRUE	ASV438.Bacteria.Proteobacteria.Gammaproteobacteria.Pseudomonadales.Moraxellaceae.Acinetobacter.NA
TRUE	TRUE	TRUE	ASV441.Bacteria.Cyanobacteria.Vampirivibrionia.Obscuribacterales.Obscuribacteraceae.Candidatus Obscuribacter.NA

TRUE	TRUE	TRUE	ASV443.Bacteria.Bacteroidota.Bacteroidia.Bacteroidales.Muribaculaceae.Muribaculaceae Unclassified.NA
FALSE	TRUE	FALSE	ASV457.Bacteria.Firmicutes.Bacilli.Brevibacillales.Brevibacillaceae.Brevibacillus.NA
FALSE	TRUE	FALSE	ASV474.Bacteria.Firmicutes.Clostridia.Lachnospirales.Lachnospiraceae.Lachnospiraceae Unclassified.NA
FALSE	TRUE	FALSE	ASV476.Bacteria.Firmicutes.Bacilli.Bacillales.Planococcaceae.Planococcaceae Unclassified.NA
FALSE	TRUE	FALSE	ASV477.Bacteria.Firmicutes.Clostridia.Clostridia UCG-014.NA.NA Unclassified.NA
FALSE	TRUE	FALSE	ASV479.Bacteria.Firmicutes.Clostridia.Lachnospirales.Lachnospiraceae.Lachnospiraceae UCG-001.NA
FALSE	TRUE	FALSE	ASV480.Bacteria.Firmicutes.Bacilli.Bacillales.NA.NA Unclassified.NA
TRUE	FALSE	FALSE	ASV488.Bacteria.Actinobacteriota.Actinobacteria.Micrococcales.Cellulomonadaceae.Cellulomonas.NA
FALSE	TRUE	FALSE	ASV489.Bacteria.Actinobacteriota.Actinobacteria.Micrococcales.Micrococcaceae.Renibacterium.NA
FALSE	TRUE	FALSE	ASV493.Bacteria.Firmicutes.Clostridia.Oscillospirales.Ruminococcaceae.Ruminococcaceae Unclassified.NA
TRUE	FALSE	FALSE	ASV504.Bacteria.Firmicutes.Bacilli.RF39.NA.NA Unclassified.NA
FALSE	TRUE	FALSE	ASV508.Bacteria.Proteobacteria.Gammaproteobacteria.Burkholderiales.Comamonadaceae.Comamonadaceae Unclassified.NA
FALSE	TRUE	FALSE	ASV510.Bacteria.Firmicutes.Bacilli.Bacillales.Bacillaceae.Bacillaceae Unclassified.NA
TRUE	TRUE	TRUE	ASV520.Bacteria.Firmicutes.Bacilli.Lactobacillales.Streptococcaceae.Streptococcus.NA
FALSE	TRUE	FALSE	ASV522.Bacteria.Proteobacteria.Gammaproteobacteria.Burkholderiales.Comamonadaceae.Tepidimonas.NA
TRUE	TRUE	TRUE	ASV532.Bacteria.Proteobacteria.Gammaproteobacteria.Pseudomonadales.Moraxellaceae.Acinetobacter.NA
TRUE	TRUE	TRUE	ASV544.Bacteria.Firmicutes.Bacilli.Lactobacillales.Streptococcaceae.Streptococcus.NA
TRUE	TRUE	TRUE	ASV558.Bacteria.Proteobacteria.Gammaproteobacteria.Burkholderiales.Burkholderiaceae.Burkholderia-Caballeronia-Paraburkholderia.NA
FALSE	TRUE	FALSE	ASV562.Bacteria.Firmicutes.Clostridia.Oscillospirales.Butyricicoccaceae.Butyricicoccaceae Unclassified.NA

FALSE	TRUE	FALSE	ASV567.Bacteria.Proteobacteria.Gammaproteobacteria.Burkholderiales.Comamonadaceae.Comamonadaceae Unclassified.NA
TRUE	TRUE	TRUE	ASV578.Bacteria.Proteobacteria.Gammaproteobacteria.Pseudomonadales.Pseudomonadaceae.Pseudomonas.NA
TRUE	FALSE	FALSE	ASV579.Bacteria.Firmicutes.Clostridia.Lachnospirales.Lachnospiraceae.Lachnospiraceae Unclassified.NA
FALSE	TRUE	FALSE	ASV590.Bacteria.Proteobacteria.Gammaproteobacteria.Enterobacterales.Morganellaceae.Proteus.NA
TRUE	TRUE	TRUE	ASV593.Bacteria.Proteobacteria.Gammaproteobacteria.Burkholderiales.Oxalobacteraceae.Massilia.NA
FALSE	TRUE	FALSE	ASV596.Bacteria.Proteobacteria.Gammaproteobacteria.Pseudomonadales.Moraxellaceae.Acinetobacter.lwoffii
FALSE	TRUE	FALSE	ASV598.Bacteria.Firmicutes.Bacilli.Bacillales.Planococcaceae.Planococcaceae Unclassified.NA
TRUE	FALSE	FALSE	ASV605.Bacteria.Firmicutes.Bacilli.Lactobacillales.Streptococcaceae.Lactococcus.NA
FALSE	TRUE	FALSE	ASV611.Bacteria.Firmicutes.Bacilli.Bacillales.Bacillaceae.Bacillus.NA
FALSE	TRUE	FALSE	ASV612.Bacteria.Firmicutes.Bacilli.Bacillales.Bacillaceae.Pseudogracilibacillus.NA
TRUE	TRUE	TRUE	ASV617.Bacteria.Proteobacteria.Alphaproteobacteria.Rhizobiales.Rhizobiaceae.Phyllobacterium.NA
FALSE	TRUE	FALSE	ASV619.Bacteria.Firmicutes.Bacilli.Bacillales.Bacillaceae.Pseudogracilibacillus.NA
TRUE	TRUE	TRUE	ASV620.Bacteria.Proteobacteria.Alphaproteobacteria.Sphingomonadales.Sphingomonadaceae.Sphingomonas.desiccabilis
TRUE	TRUE	TRUE	ASV627.Bacteria.Proteobacteria.Alphaproteobacteria.Caulobacterales.Caulobacteraceae.Brevundimonas.NA
TRUE	FALSE	FALSE	ASV631.Bacteria.Firmicutes.Bacilli.Lactobacillales.Lactobacillaceae.Lactobacillus.NA
TRUE	FALSE	FALSE	ASV632.Bacteria.Firmicutes.Clostridia.Lachnospirales.Lachnospiraceae.Lachnospiraceae Unclassified.NA
FALSE	TRUE	FALSE	ASV634.Bacteria.Firmicutes.Bacilli.Bacillales.Bacillaceae.Bacillaceae Unclassified.NA
TRUE	FALSE	FALSE	ASV636.Bacteria.Firmicutes.Clostridia.Clostridia UCG-014.NA.NA Unclassified.NA
TRUE	TRUE	TRUE	ASV637.Bacteria.Proteobacteria.Gammaproteobacteria.Burkholderiales.Comamonadaceae.Comamonadaceae Unclassified.NA

TRUE	TRUE	TRUE	ASV638.Bacteria.Actinobacteriota.Actinobacteria.Corynebacteriales.Nocardiaceae.Rhodococcus.NA
TRUE	FALSE	FALSE	ASV639.Bacteria.Firmicutes.Clostridia.Lachnospirales.Lachnospiraceae.Lachnospiraceae UCG-001.NA
FALSE	TRUE	FALSE	ASV645.Bacteria.Proteobacteria.Alphaproteobacteria.Sphingomonadales.Sphingomonadaceae.Sphingobium.NA
TRUE	TRUE	TRUE	ASV651.Bacteria.Proteobacteria.Alphaproteobacteria.Rhizobiales.Xanthobacteraceae.Bradyrhizobium.NA
FALSE	TRUE	FALSE	ASV654.Bacteria.Deinococcota.Deinococci.Thermales.Thermaceae.Thermus.NA
FALSE	TRUE	FALSE	ASV656.Bacteria.Firmicutes.Clostridia.Lachnospirales.Lachnospiraceae.Lachnospiraceae Unclassified.NA
FALSE	TRUE	FALSE	ASV661.Bacteria.Firmicutes.Bacilli.Bacillales.Bacillaceae.Pseudogracilibacillus.NA
TRUE	FALSE	FALSE	ASV662.Bacteria.Firmicutes.Clostridia.Clostridia UCG-014.NA.NA Unclassified.NA
FALSE	TRUE	FALSE	ASV666.Bacteria.Firmicutes.Bacilli.Bacillales.NA.NA Unclassified.NA
FALSE	TRUE	FALSE	ASV671.Bacteria.Proteobacteria.Gammaproteobacteria.Pseudomonadales.Moraxellaceae.Acinetobacter.NA
FALSE	TRUE	FALSE	ASV686.Bacteria.Proteobacteria.Gammaproteobacteria.Pseudomonadales.Pseudomonadaceae.Pseudomonas.NA
FALSE	TRUE	FALSE	ASV693.Bacteria.Firmicutes.Clostridia.Clostridiales.Clostridiaceae.Clostridium sensu stricto 1.isatidis
FALSE	TRUE	FALSE	ASV694.Bacteria.Proteobacteria.Gammaproteobacteria.Pasteurellales.Pasteurellaceae.Haemophilus.NA
FALSE	TRUE	FALSE	ASV696.Bacteria.Firmicutes.Limnochordia.Limnochordales.Limnochordaceae.Limnochordaceae Unclassified.NA
FALSE	TRUE	FALSE	ASV702.Bacteria.Proteobacteria.Alphaproteobacteria.Rhodobacterales.Rhodobacteraceae.Rhodobacteraceae Unclassified.NA
TRUE	TRUE	TRUE	ASV708.Bacteria.Proteobacteria.Gammaproteobacteria.Burkholderiales.Comamonadaceae.Paucibacter.NA
FALSE	TRUE	FALSE	ASV710.Bacteria.Firmicutes.Bacilli.Bacillales.Bacillaceae.Anoxybacillus.NA
TRUE	FALSE	FALSE	ASV717.Bacteria.Proteobacteria.Alphaproteobacteria.Caulobacterales.Caulobacteraceae.Caulobacteraceae Unclassified.NA
TRUE	FALSE	FALSE	ASV720.Bacteria.Firmicutes.Clostridia.Clostridia UCG-014.NA.NA Unclassified.NA

FALSE TRUE FALSE ASV721.Bacteria.Proteobacteria.Gammaproteobacteria.Burkholderiales.Comamonadaceae.Comamonadaceae Unclassified.NA

TRUE FALSE FALSE ASV727.Bacteria.Proteobacteria.Gammaproteobacteria.Enterobacterales.Enterobacteriaceae.Enterobacteriaceae Unclassified.NA

FALSE TRUE FALSE ASV728.Bacteria.Proteobacteria.Gammaproteobacteria.Xanthomonadales.Xanthomonadaceae.Stenotrophomonas.acidaminiphila

FALSE TRUE FALSE ASV730.Bacteria.Actinobacteriota.Actinobacteria.Corynebacteriales.Corynebacteriaceae.Corynebacterium.NA

TRUE FALSE FALSE ASV734.Bacteria.Actinobacteriota.Actinobacteria.Frankiales.NA.NA Unclassified.NA

FALSE TRUE FALSE ASV739.Bacteria.Firmicutes.Bacilli.Brevibacillales.Brevibacillaceae.Brevibacillus.NA

TRUE FALSE FALSE ASV741.Bacteria.Proteobacteria.Alphaproteobacteria.Sphingomonadales.Sphingomonadaceae.Sphingomonadaceae Unclassified.NA

TRUE TRUE TRUE ASV744.Bacteria.Proteobacteria.Gammaproteobacteria.Burkholderiales.Comamonadaceae.Comamonadaceae Unclassified.NA

FALSE TRUE FALSE ASV753.Bacteria.Proteobacteria.Gammaproteobacteria.Xanthomonadales.Xanthomonadaceae.Lysobacter.NA

FALSE TRUE FALSE ASV758.Bacteria.Firmicutes.Bacilli.Paenibacillales.Paenibacillaceae.Paenibacillus.NA

FALSE TRUE FALSE ASV762.Bacteria.Firmicutes.Negativicutes.Veillonellales-Selenomonadales.Veillonellaceae.Veillonella.NA

FALSE TRUE FALSE ASV771.Bacteria.Proteobacteria.Gammaproteobacteria.Pseudomonadales.Moraxellaceae.Acinetobacter.NA

FALSE TRUE FALSE ASV776.Bacteria.Proteobacteria.Gammaproteobacteria.Pseudomonadales.Moraxellaceae.Enhydrobacter.NA

FALSE TRUE FALSE ASV780.Bacteria.Firmicutes.Clostridia.Clostridiales.Clostridiaceae.Clostridium sensu stricto 1.NA

FALSE TRUE FALSE ASV782.Bacteria.Firmicutes.Bacilli.Exiguobacterales.Exiguobacteraceae.Exiguobacterium.NA

FALSE TRUE FALSE ASV784.Bacteria.Actinobacteriota.Actinobacteria.Micrococcales.Micrococcaceae.Micrococcus.NA

FALSE TRUE FALSE ASV802.Bacteria.Proteobacteria.Gammaproteobacteria.Cellvibrionales.Cellvibrionaceae.Cellvibrio.NA

TRUE	FALSE	FALSE	ASV805.Bacteria.Firmicutes.Bacilli.Lactobacillales.Streptococcaceae.Streptococcus.NA
FALSE	TRUE	FALSE	ASV812.Bacteria.Bdellovibrionota.Oligoflexia.0319-6G20.NA.NA Unclassified.NA
TRUE	FALSE	FALSE	ASV816.Bacteria.Actinobacteriota.Actinobacteria.Micrococcales.Microbacteriaceae.Microbacteriaceae Unclassified.NA
FALSE	TRUE	FALSE	ASV822.Bacteria.Firmicutes.Bacilli.NA.NA.NA Unclassified.NA
FALSE	TRUE	FALSE	ASV823.Bacteria.Proteobacteria.Gammaproteobacteria.Burkholderiales.Comamonadaceae.Comamonas.NA
FALSE	TRUE	FALSE	ASV824.Bacteria.Actinobacteriota.Actinobacteria.Corynebacteriales.Corynebacteriaceae.Corynebacterium.NA
TRUE	FALSE	FALSE	ASV825.Bacteria.Firmicutes.Clostridia.Clostridia UCG-014.NA.NA Unclassified.NA
FALSE	TRUE	FALSE	ASV832.Bacteria.Firmicutes.Bacilli.Bacillales.Planococcaceae.Sporosarcina.NA
FALSE	TRUE	FALSE	ASV833.Bacteria.Proteobacteria.Gammaproteobacteria.Pseudomonadales.Pseudomonadaceae.Pseudomonas.NA
FALSE	TRUE	FALSE	ASV836.Bacteria.Proteobacteria.Alphaproteobacteria.Caulobacterales.Caulobacteraceae.Brevundimonas.NA
TRUE	TRUE	TRUE	ASV844.Bacteria.Actinobacteriota.Actinobacteria.Corynebacteriales.Corynebacteriaceae.Lawsonella.clevelandensis
FALSE	TRUE	FALSE	ASV847.Bacteria.Proteobacteria.Gammaproteobacteria.Enterobacterales.Morganellaceae.Providencia.NA
FALSE	TRUE	FALSE	ASV854.Bacteria.Proteobacteria.Gammaproteobacteria.Burkholderiales.Comamonadaceae.Comamonadaceae Unclassified.NA
FALSE	TRUE	FALSE	ASV855.Bacteria.Actinobacteriota.Actinobacteria.Frankiales.Geodermatophilaceae.Blastococcus.NA
FALSE	TRUE	FALSE	ASV860.Bacteria.Firmicutes.Bacilli.NA.NA.NA Unclassified.NA
TRUE	FALSE	FALSE	ASV862.Bacteria.Firmicutes.Negativicutes.Veillonellales-Selenomonadales.NA.NA Unclassified.NA
FALSE	TRUE	FALSE	ASV868.Bacteria.Actinobacteriota.Actinobacteria.Micrococcales.Micrococcaceae.Garicola.NA
FALSE	TRUE	FALSE	ASV869.Bacteria.Bacteroidota.Bacteroidia.Cytophagales.Microscillaceae.Flexibacter.NA
FALSE	TRUE	FALSE	ASV874.Bacteria.Firmicutes.Bacilli.Bacillales.Planococcaceae.Planococcaceae Unclassified.NA
FALSE	TRUE	FALSE	ASV877.Bacteria.Proteobacteria.Alphaproteobacteria.Caulobacterales.Caulobacteraceae.Brevundimonas.NA

FALSE TRUE FALSE ASV882.Bacteria.Proteobacteria.Alphaproteobacteria.Rhizobiales.Rhizobiaceae.Rhizobiaceae Unclassified.NA

FALSE TRUE FALSE ASV886.Bacteria.Actinobacteriota.Actinobacteria.Micrococcales.Micrococcaceae.Kocuria.NA

TRUE FALSE FALSE ASV895.Bacteria.Firmicutes.Clostridia.Oscillospirales.[Eubacterium] coprostanoligenes group.[Eubacterium] coprostanoligenes group Unclassified.NA

FALSE TRUE FALSE ASV898.Bacteria.Firmicutes.Bacilli.Bacillales.Bacillaceae.Bacillaceae Unclassified.NA

FALSE TRUE FALSE ASV901.Bacteria.Firmicutes.Bacilli.Aneurinibacillales.Aneurinibacillaceae.Aneurinibacillus.thermoaerophilus

TRUE FALSE FALSE ASV903.Bacteria.Firmicutes.Clostridia.Clostridia UCG-014.NA.NA Unclassified.NA

FALSE TRUE FALSE ASV922.Bacteria.Firmicutes.Bacilli.Bacillales.Planococcaceae.Sporosarcina.soli

FALSE TRUE FALSE ASV927.Bacteria.Proteobacteria.Gammaproteobacteria.Burkholderiales.Alcaligenaceae.Alcaligenes.NA

FALSE TRUE FALSE ASV930.Bacteria.Firmicutes.Bacilli.Bacillales.Planococcaceae.Sporosarcina.NA

TRUE FALSE FALSE ASV946.Bacteria.Firmicutes.Clostridia.Oscillospirales.Ruminococcaceae.Faecalibacterium.prausnitzii

TRUE FALSE FALSE ASV957.Bacteria.Firmicutes.Clostridia.Lachnospirales.Lachnospiraceae.Lachnospiraceae Unclassified.NA

TRUE FALSE FALSE ASV966.Bacteria.Firmicutes.Clostridia.Clostridia UCG-014.NA.NA Unclassified.NA

FALSE TRUE FALSE ASV978.Bacteria.Proteobacteria.Gammaproteobacteria.Xanthomonadales.Xanthomonadaceae.Stenotrophomonas.NA

TRUE FALSE FALSE ASV994.Bacteria.Firmicutes.Clostridia.Clostridia vadinBB60 group.NA.NA Unclassified.NA

FALSE TRUE FALSE ASV995.Bacteria.Firmicutes.Bacilli.Bacillales.Bacillaceae.Bacillaceae Unclassified.NA

FALSE TRUE FALSE ASV996.Bacteria.Proteobacteria.Alphaproteobacteria.Rhizobiales.Beijerinckiaceae.Methylobacterium-Methylorubrum.NA

FALSE TRUE FALSE ASV997.Bacteria.Proteobacteria.Gammaproteobacteria.Alteromonadales.Alteromonadaceae.Alishewanella.NA

FALSE TRUE FALSE ASV1005.Bacteria.Actinobacteriota.Actinobacteria.Frankiales.Geodermatophilaceae.Blastococcus.NA

FALSE TRUE FALSE ASV1007.Bacteria.Proteobacteria.Gammaproteobacteria.Burkholderiales.Rhodocyclaceae.Rhodocyclaceae Unclassified.NA

TRUE	FALSE	FALSE	ASV1026.Bacteria.Actinobacteriota.Actinobacteria.Micrococcales.Brevibacteriaceae.Brevibacterium.NA
FALSE	TRUE	FALSE	ASV1029.Bacteria.Proteobacteria.Gammaproteobacteria.Burkholderiales.Alcaligenaceae.Paenalcaligenes.NA
FALSE	TRUE	FALSE	ASV1030.Bacteria.Bacteroidota.Bacteroidia.Flavobacteriales.Weeksellaceae.Moheibacter.NA
FALSE	TRUE	FALSE	ASV1031.Bacteria.Deinococcota.Deinococci.Deinococcales.Deinococcaceae.Deinococcus.NA
FALSE	TRUE	FALSE	ASV1051.Bacteria.Proteobacteria.Gammaproteobacteria.Burkholderiales.Alcaligenaceae.Achromobacter.NA
FALSE	TRUE	FALSE	ASV1054.Bacteria.Bacteroidota.Bacteroidia.Cytophagales.Microscillaceae.Microscillaceae Unclassified.NA
FALSE	TRUE	FALSE	ASV1057.Bacteria.Firmicutes.Bacilli.Bacillales.Bacillaceae.Bacillaceae Unclassified.NA
TRUE	FALSE	FALSE	ASV1071.Bacteria.Bacteroidota.Bacteroidia.Bacteroidales.Bacteroidaceae.Bacteroides.NA
TRUE	FALSE	FALSE	ASV1083.Bacteria.Firmicutes.Clostridia.Clostridia UCG-014.NA.NA Unclassified.NA
FALSE	TRUE	FALSE	ASV1084.Bacteria.Proteobacteria.Gammaproteobacteria.Pseudomonadales.Pseudomonadaceae.Pseudomonas.NA
FALSE	TRUE	FALSE	ASV1088.Bacteria.Actinobacteriota.Actinobacteria.Pseudonocardiales.Pseudonocardiaceae.Pseudonocardia.NA
FALSE	TRUE	FALSE	ASV1092.Bacteria.Proteobacteria.Gammaproteobacteria.Burkholderiales.Sutterellaceae.AAP99.NA
FALSE	TRUE	FALSE	ASV1099.Bacteria.Proteobacteria.Gammaproteobacteria.Enterobacterales.Yersiniaceae.Yersinia.NA
FALSE	TRUE	FALSE	ASV1107.Bacteria.Firmicutes.Bacilli.Lactobacillales.Lactobacillaceae.Lactobacillus.NA
FALSE	TRUE	FALSE	ASV1111.Bacteria.Proteobacteria.Gammaproteobacteria.Xanthomonadales.Xanthomonadaceae.Xanthomonadaceae Unclassified.NA
FALSE	TRUE	FALSE	ASV1130.Bacteria.Proteobacteria.Gammaproteobacteria.Burkholderiales.Burkholderiaceae.Burkholderia-Caballeronia-Paraburkholderia.NA
TRUE	FALSE	FALSE	ASV1132.Bacteria.Actinobacteriota.Actinobacteria.Micrococcales.Micrococcaceae.Rothia.dentocariosa
TRUE	FALSE	FALSE	ASV1168.Bacteria.Bacteroidota.Bacteroidia.Bacteroidales.Muribaculaceae.Muribaculaceae Unclassified.NA
TRUE	FALSE	FALSE	ASV1187.Bacteria.Firmicutes.Clostridia.Lachnospirales.Lachnospiraceae.Lachnospiraceae UCG-004.NA

TRUE	FALSE	FALSE	ASV1197.Bacteria.Firmicutes.Clostridia.Lachnospirales.Lachnospiraceae.Lachnospiraceae Unclassified.NA
TRUE	FALSE	FALSE	ASV1199.Bacteria.Bacteroidota.Bacteroidia.Bacteroidales.Muribaculaceae.Muribaculaceae Unclassified.NA
TRUE	FALSE	FALSE	ASV1206.Bacteria.Firmicutes.Bacilli.Erysipelotrichales.Erysipelotrichaceae.Faecalibaculum.NA
TRUE	FALSE	FALSE	ASV1211.Bacteria.Bacteroidota.Bacteroidia.NA.NA.NA Unclassified.NA

Supplemental Figure 1. Results comparing the influence of atmosphere on the cultured microbiota of the oral cavity of 11 pregnant mice. In panels A and B, principal coordinates analysis plots indicate bacterial composition (A) and structure (B) of the oral cavity cultured under anoxic, hypoxic, and oxic atmospheres. Ellipses indicate standard deviation for each atmosphere and in panel B, ASVs with the highest relative average abundances were plotted using weighted average scores calculated with the vegan package. The heatmap in panel C includes ASVs with ≥ 1% average relative abundance and samples are clustered by Bray-Curtis similarities within each atmosphere. The cladogram in panel D illustrates LEfSe results of taxa preferentially recovered under one atmosphere over the others. Each node represents a taxon at each taxonomic level starting with the kingdom Bacteria down through genus in the outermost nodes and are colored based on preferential recovery under a specific atmosphere. Yellow nodes represent taxa that were not recovered preferentially under a particular atmosphere. The diameter of each node is proportional to the relative abundance of that taxa. Phyla, class, and order (not labeled) clades are highlighted when significant for a particular atmosphere. Statistical comparisons were performed using non-parametric MANOVAs with the vegan package in R (v. 4.0.3).

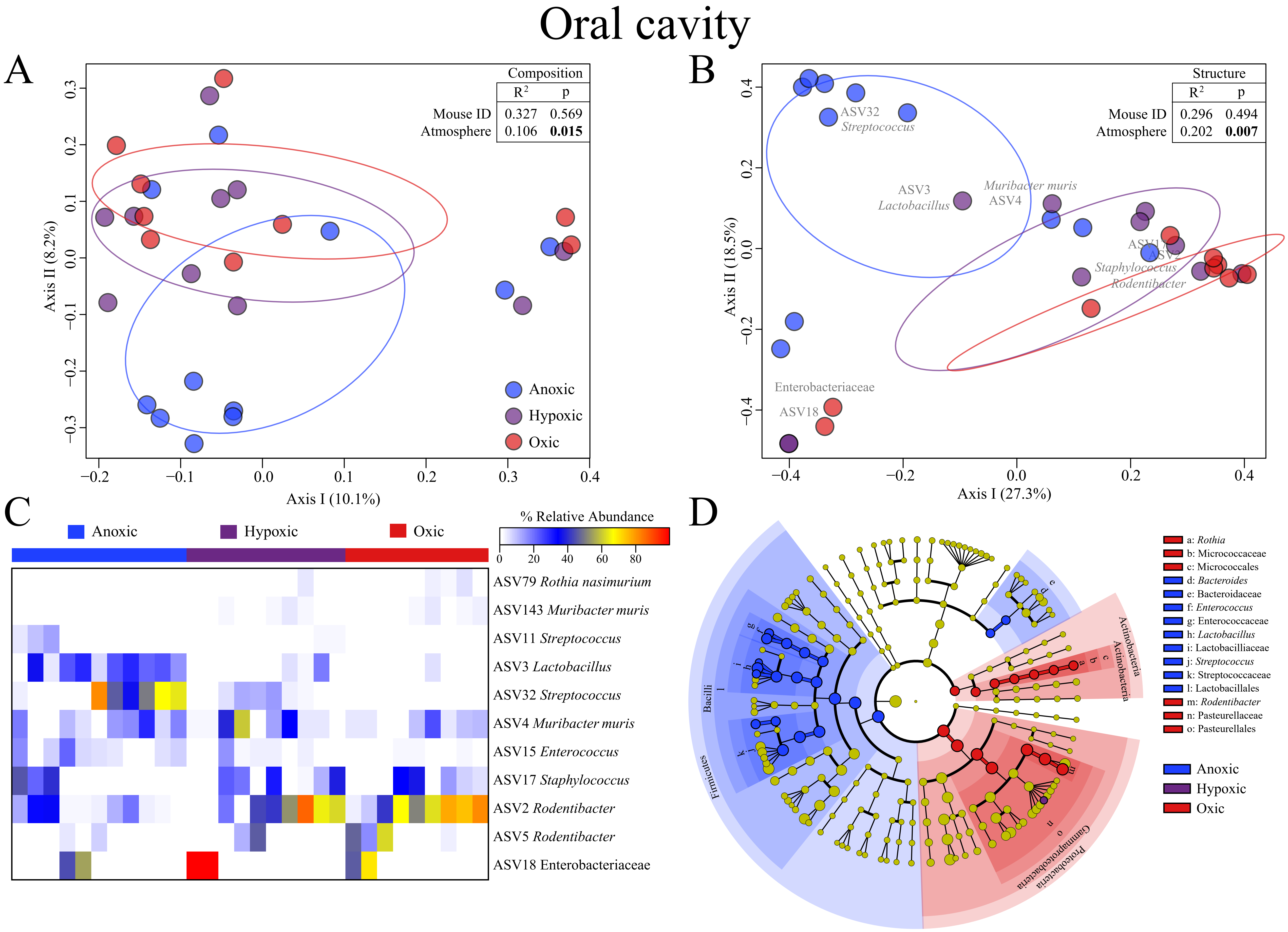

Oral cavity
A
Composition
R²
p
Mouse ID 0.327 0.569
Atmosphere 0.106 0.015
Axis II (8.2%)
Axis I (10.1%)
Anoxic
Hypoxic
Oxic
B
Structure
R²
p
Mouse ID 0.296 0.494
Atmosphere 0.202 0.007
ASV32 Streptococcus
ASV3 Lactobacillus
Muribacter muris ASV4
Staphylococcus
Rodentibacter
Enterobacteriaceae
ASV18
Axis II (18.5%)
Axis I (27.3%)
C
Anoxic
Hypoxic
Oxic
% Relative Abundance
0 20 40 60 80
ASV79 Rothia nasimurium
ASV143 Muribacter muris
ASV11 Streptococcus
ASV3 Lactobacillus
ASV32 Streptococcus
ASV4 Muribacter muris
ASV15 Enterococcus
ASV17 Staphylococcus
ASV2 Rodentibacter
ASV5 Rodentibacter
ASV18 Enterobacteriaceae
D
a: Rothia
b: Micrococcaceae
c: Micrococcales
d: Bacteroides
e: Bacteroidaceae
f: Enterococcus
g: Enterococcaceae
h: Lactobacillus
i: Lactobacilliaceae
j: Streptococcus
k: Streptococcaceae
l: Lactobacillales
m: Rodentibacter
n: Pasteurellaceae
o: Pasteurellales
Anoxic
Hypoxic
Oxic
Actinobacteria
Bacilli
Firmicutes
Gammaproteobacteria
Proteobacteria

Supplemental Figure 2. Results comparing the influence of atmosphere on the cultured microbiota of the lung of 10 pregnant mice. In panels A and B, principal coordinates analysis plots indicate bacterial composition (A) and structure (B) of the lung cultured under anoxic, hypoxic, and oxic atmospheres. The heatmap in panel C includes ASVs with ≥ 1% average relative abundance and samples are clustered by Bray-Curtis similarities. Statistical comparisons were performed using non-parametric MANOVAs with the vegan package in R (v. 4.0.3).

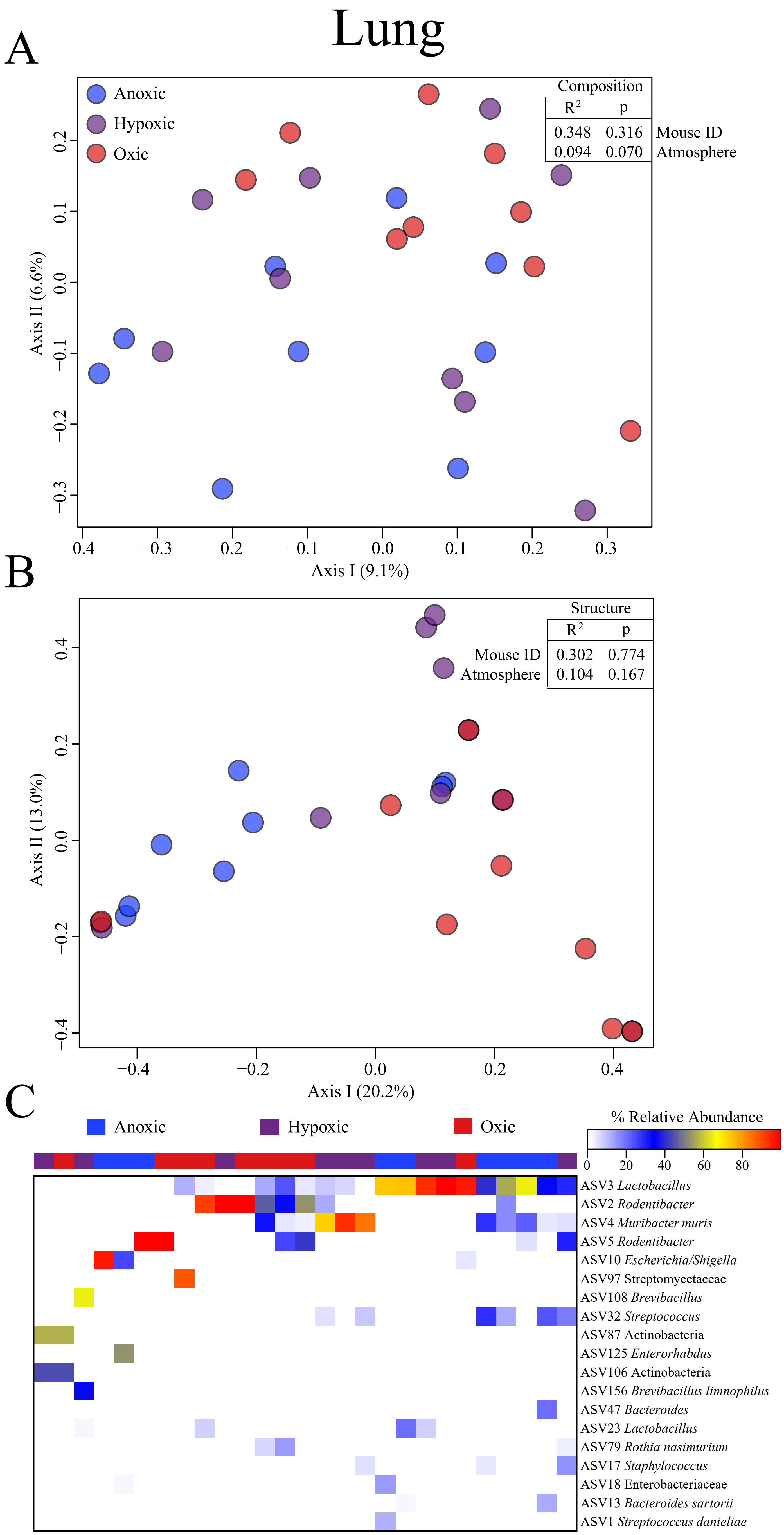

Lung
A
Anoxic
Hypoxic
Oxic
Composition
R2 p
0.348 0.316 Mouse ID
0.094 0.070 Atmosphere
Axis II (6.6%)
Axis I (9.1%)
B
Structure
R2 p
Mouse ID 0.302 0.774
Atmosphere 0.104 0.167
Axis II (13.0%)
Axis I (20.2%)
C
% Relative Abundance
ASV3 Lactobacillus
ASV2 Rodentibacter
ASV4 Muribacter muris
ASV5 Rodentibacter
ASV10 Escherichia/Shigella
ASV97 Streptomycetaceae
ASV108 Brevibacillus
ASV32 Streptococcus
ASV87 Actinobacteria
ASV125 Enterorhabdus
ASV106 Actinobacteria
ASV156 Brevibacillus limnophilus
ASV47 Bacteroides
ASV23 Lactobacillus
ASV79 Rothia nasimurium
ASV17 Staphylococcus
ASV18 Enterobacteriaceae
ASV13 Bacteroides sartorii
ASV1 Streptococcus danieliae

Supplemental Figure 3. Results comparing the influence of atmosphere on the cultured microbiota of the intestine of 11 pregnant mice. In panels A and B, principal coordinates analysis plots indicate bacterial composition (A) and structure (B) of the intestine cultured under anoxic, hypoxic, and oxic atmospheres. Ellipses indicate standard deviation for each atmosphere and in panel B, ASVs with the highest relative average abundances were plotted using weighted average scores calculated with the vegan package. The heatmap in panel C includes ASVs with $\geq$ 1% average relative abundance and samples are clustered by Bray-Curtis similarities within each atmosphere. The cladogram in panel D illustrates LEfSe results of taxa preferentially recovered under one atmosphere over the others. Each node represents a taxon at each taxonomic level starting with the kingdom Bacteria down through genus in the outermost nodes and are colored based on preferential recovery under a specific atmosphere. Yellow nodes represent taxa that were not recovered preferentially under a particular atmosphere. The diameter of each node is proportional to the relative abundance of that taxa. Phyla, class, and order (not labeled) clades are highlighted when significant for a particular atmosphere. Statistical comparisons were performed using non-parametric MANOVAs with the vegan package in R (v. 4.0.3).

Intestine

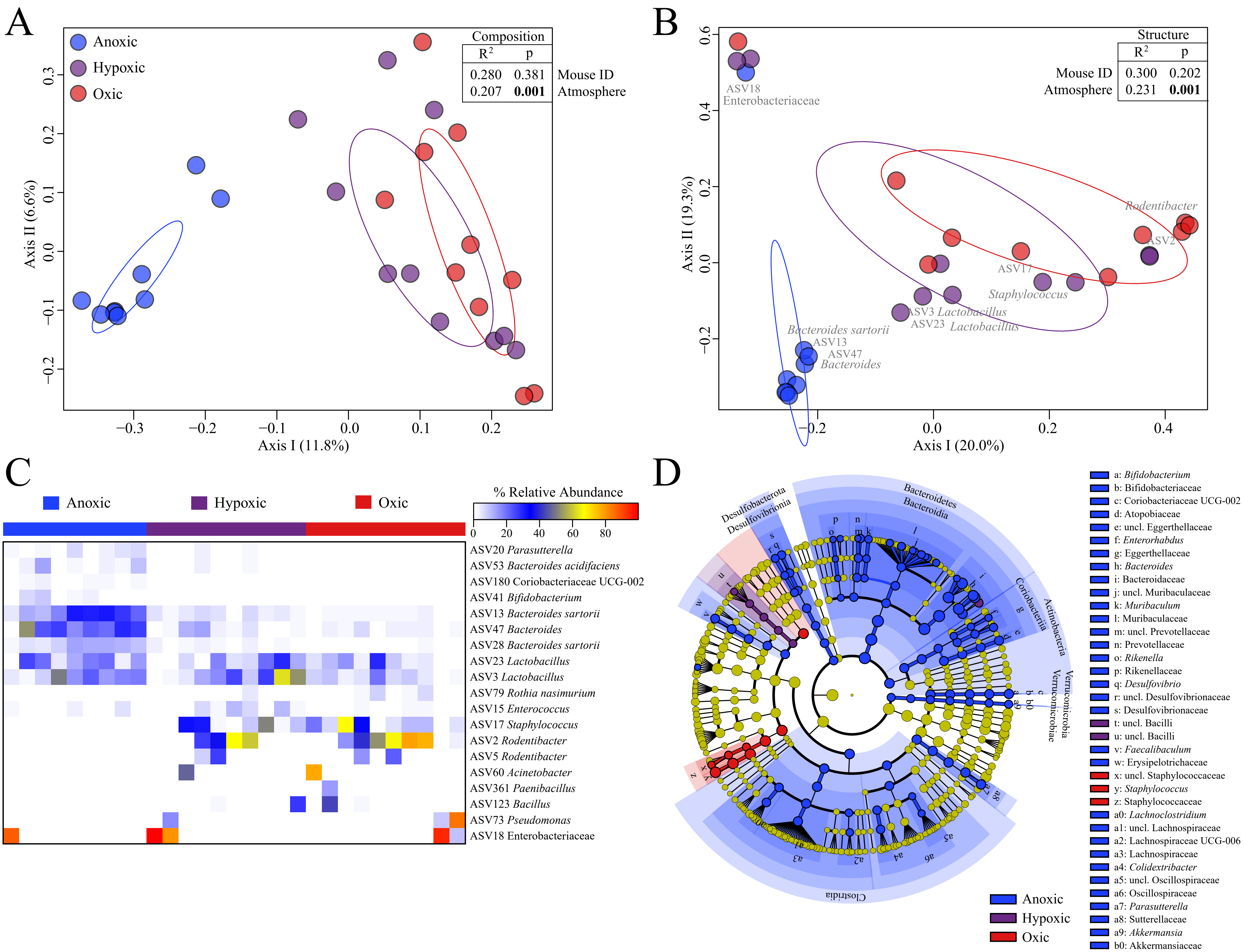
A
Anoxic
Hypoxic
Oxic
Composition
R2 p
0.280 0.381 Mouse ID
0.207 0.001 Atmosphere
Axis II (6.6%)
Axis I (11.8%)
B
Structure
R2 p
Mouse ID 0.300 0.202
Atmosphere 0.231 0.001
ASV18
Enterobacteriaceae
Rodentibacter
ASV2
ASV17
Staphylococcus
ASV3 Lactobacillus
ASV23 Lactobacillus
Bacteroides sartorii
ASV13
ASV47
Bacteroides
Axis II (19.3%)
Axis I (20.0%)
C
Anoxic
Hypoxic
Oxic
% Relative Abundance
0 20 40 60 80
ASV20 Parasutterella
ASV53 Bacteroides acidifaciens
ASV180 Coriobacteriaceae UCG-002
ASV41 Bifidobacterium
ASV13 Bacteroides sartorii
ASV47 Bacteroides
ASV28 Bacteroides sartorii
ASV23 Lactobacillus
ASV3 Lactobacillus
ASV79 Rothia nasimurium
ASV15 Enterococcus
ASV17 Staphylococcus
ASV2 Rodentibacter
ASV5 Rodentibacter
ASV60 Acinetobacter
ASV361 Paenibacillus
ASV123 Bacillus
ASV73 Pseudomonas
ASV18 Enterobacteriaceae
D
Desulfobacterota
Desulfovibrionia
Bacteroidetes
Bacteroidia
Coriobacteriia
Actinobacteria
Verrucomicrobia
Verrucomicrobiae
Clostridia
a: Bifidobacterium
b: Bifidobacteriaceae
c: Coriobacteriaceae UCG-002
d: Atopobiaceae
e: uncl. Eggerthellaceae
f: Enterorhabdus
g: Eggerthellaceae
h: Bacteroides
i: Bacteroidaceae
j: uncl. Muribaculaceae
k: Muribaculum
l: Muribaculaceae
m: uncl. Prevotellaceae
n: Prevotellaceae
o: Rikenella
p: Rikenellaceae
q: Desulfovibrio
r: uncl. Desulfovibrionaceae
s: Desulfovibrionaceae
t: uncl. Bacilli
u: uncl. Bacilli
v: Faecalibaculum
w: Erysipelotrichaceae
x: uncl. Staphylococcaceae
y: Staphylococcus
z: Staphylococcaceae
a0: Lachnoclostridium
a1: uncl. Lachnospiraceae
a2: Lachnospiraceae UCG-006
a3: Lachnospiraceae
a4: Colidextribacter
a5: uncl. Oscillospiraceae
a6: Oscillospiraceae
a7: Parasutterella
a8: Sutterellaceae
a9: Akkermansia
b0: Akkermansiaceae
Anoxic
Hypoxic
Oxic

Supplemental Figure 4. Results comparing the influence of atmosphere on the cultured microbiota of the vagina of 11 pregnant mice. In panels A and B, principal coordinates analysis plots indicate bacterial composition (A) and structure (B) of the vagina cultured under anoxic, hypoxic, and oxic atmospheres with mouse identity indicated by letters A through K because mouse identity was a statistically significant driver of variation in this body site. In panel B, ASVs with the highest relative average abundances were plotted using weighted average scores calculated with the vegan package. The heatmap in panel C includes ASVs with $\geq$ 1% average relative abundance and samples are clustered by Bray-Curtis similarities. Statistical comparisons were performed using non-parametric MANOVAs with the vegan package in R (v. 4.0.3).

Vagina

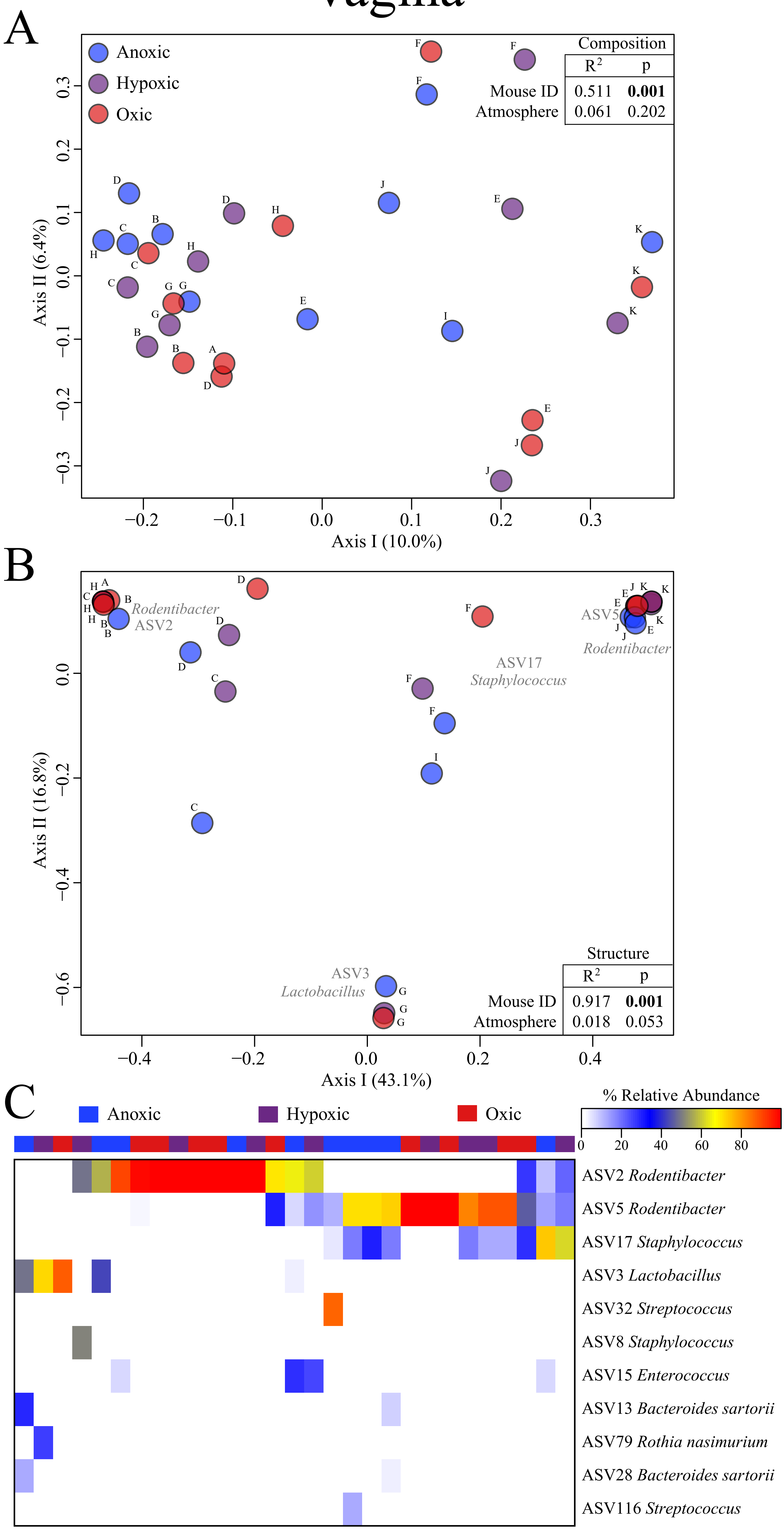
A
Anoxic
Hypoxic
Oxic
Composition
R²
p
Mouse ID
0.511
0.001
Atmosphere
0.061
0.202
Axis I (10.0%)
Axis II (6.4%)
B
Rodentibacter
ASV2
ASV5
Rodentibacter
ASV17
Staphylococcus
ASV3
Lactobacillus
Structure
R²
p
Mouse ID
0.917
0.001
Atmosphere
0.018
0.053
Axis I (43.1%)
Axis II (16.8%)
C
Anoxic
Hypoxic
Oxic
% Relative Abundance
0
20
40
60
80
ASV2 Rodentibacter
ASV5 Rodentibacter
ASV17 Staphylococcus
ASV3 Lactobacillus
ASV32 Streptococcus
ASV8 Staphylococcus
ASV15 Enterococcus
ASV13 Bacteroides sartorii
ASV79 Rothia nasimurium
ASV28 Bacteroides sartorii
ASV116 Streptococcus

Supplemental Figure 5. LEfSe results identifying individual ASVs recovered preferentially under one atmosphere over the others for the oral cavity, lung, and intestine. For each panel Linear discriminate analysis Effect Size (LEfSe) analysis was performed on ASVs cultured from the indicated boy site to identify ASVs preferentially recovered under one atmospheric condition over the others. No ASVs were preferentially recovered under hypoxic or oxic conditions in the lung. No individual ASVs were identified as significant by LEfSe for vaginal samples under any atmosphere.

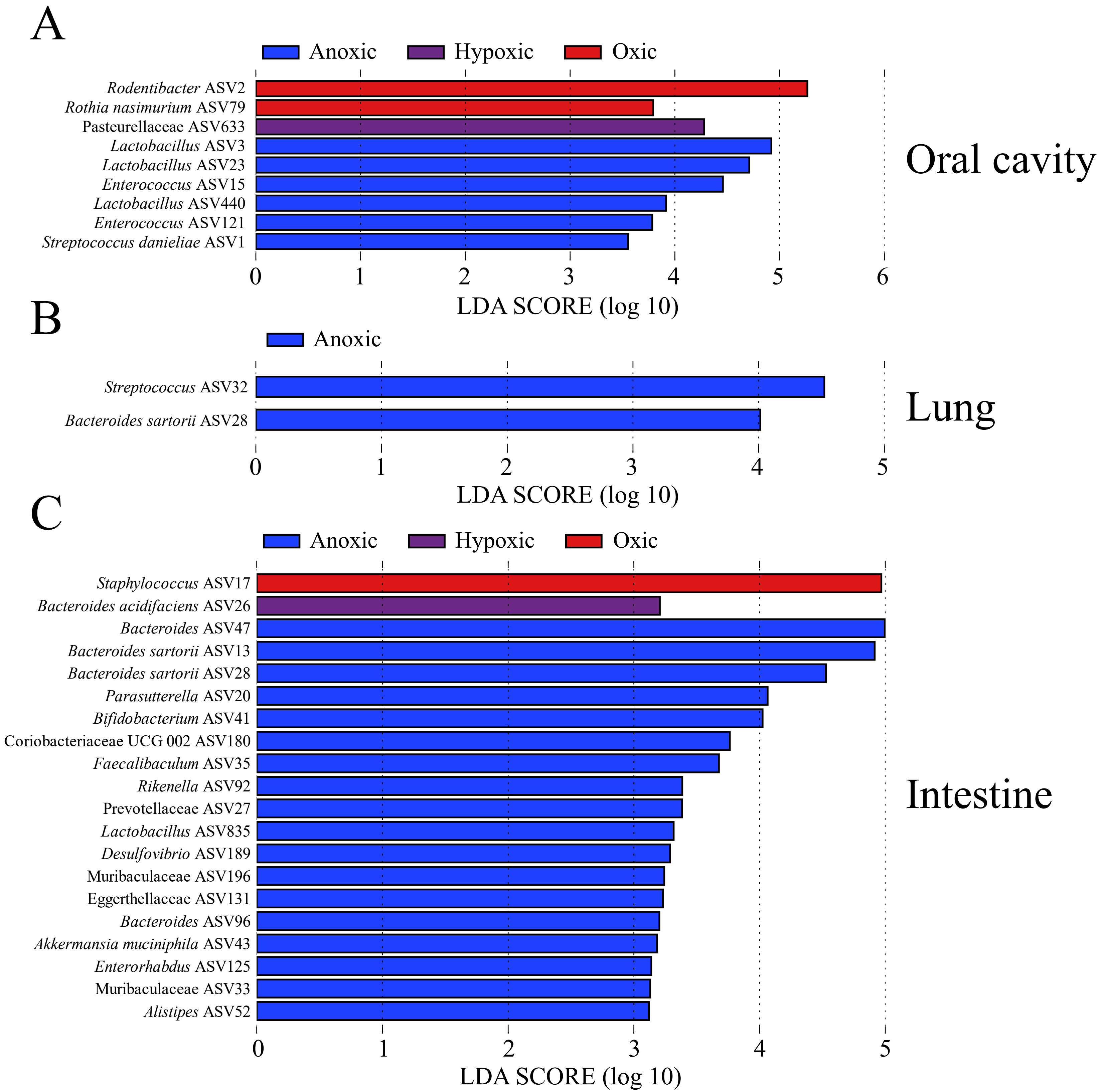
A
Anoxic
Hypoxic
Oxic
Rodentibacter ASV2
Rothia nasimurium ASV79
Pasteurellaceae ASV633
Lactobacillus ASV3
Lactobacillus ASV23
Enterococcus ASV15
Lactobacillus ASV440
Enterococcus ASV121
Streptococcus danieliae ASV1
0 1 2 3 4 5 6
LDA SCORE (log 10)
Oral cavity
B
Anoxic
Streptococcus ASV32
Bacteroides sartorii ASV28
0 1 2 3 4 5
LDA SCORE (log 10)
Lung
C
Anoxic
Hypoxic
Oxic
Staphylococcus ASV17
Bacteroides acidifaciens ASV26
Bacteroides ASV47
Bacteroides sartorii ASV13
Bacteroides sartorii ASV28
Parasutterella ASV20
Bifidobacterium ASV41
Coriobacteriaceae UCG 002 ASV180
Faecalibaculum ASV35
Rikenella ASV92
Prevotellaceae ASV27
Lactobacillus ASV835
Desulfovibrio ASV189
Muribaculaceae ASV196
Eggerthellaceae ASV131
Bacteroides ASV96
Akkermansia muciniphila ASV43
Enterorhabdus ASV125
Muribaculaceae ASV33
Alistipes ASV52
0 1 2 3 4 5
LDA SCORE (log 10)
Intestine

Supplemental Figure 6. Graphical illustration of the glycogen metabolism pathway encoded in the sequenced genomes of *Rodentibacter pneumotropicus* and *R. heylii* isolates ASV2 and ASV5, respectively. In panel A, the glycogen metabolism pathway is indicated with enzymes and enzyme commission numbers (ECs) along with the specific gene locus encoded in each genome (locus tag prefix MUU45 for *R. pneumotropicus* and MUW69 for *R. heylii)*. In panel B and C, the encoded genes corresponding to the pathway in panel A are represented illustrating their orientations in the genomes of each isolate. Gene names are color-coded by isolate (blue for *R. pneumotropicus* and green for *R. heylii*). Glycogen pathway and gene representations were generated using Pathway Tools (v 25.0).

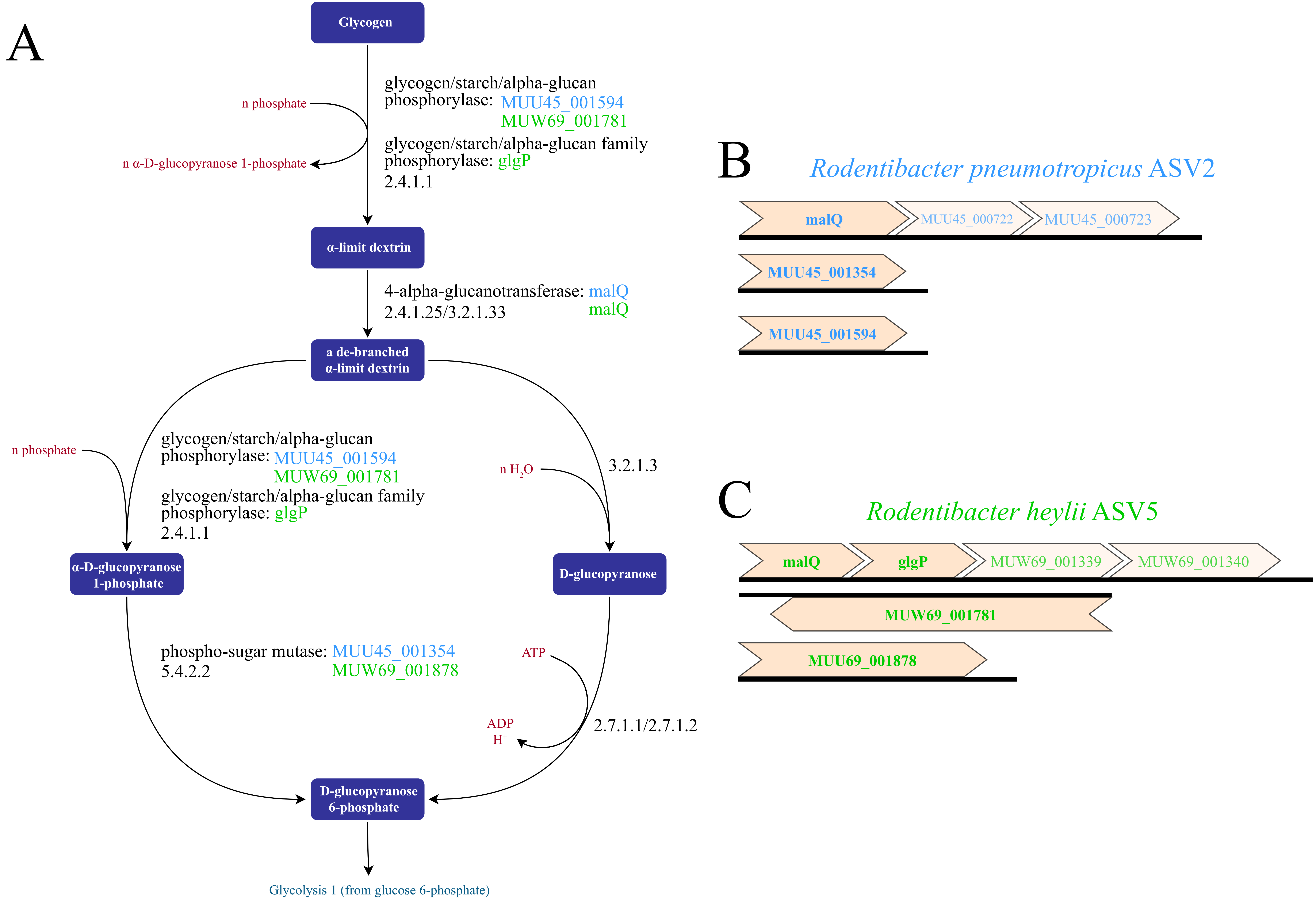

A
Glycogen
glycogen/starch/alpha-glucan phosphorylase: MUU45_001594 MUW69_001781
glycogen/starch/alpha-glucan family phosphorylase: glgP
2.4.1.1
n phosphate
n α-D-glucopyranose 1-phosphate
α-limit dextrin
4-alpha-glucanotransferase: malQ malQ
2.4.1.25/3.2.1.33
a de-branched α-limit dextrin
glycogen/starch/alpha-glucan phosphorylase: MUU45_001594 MUW69_001781
glycogen/starch/alpha-glucan family phosphorylase: glgP
2.4.1.1
n phosphate
n H_2O
3.2.1.3
α-D-glucopyranose 1-phosphate
D-glucopyranose
phospho-sugar mutase: MUU45_001354 MUW69_001878
5.4.2.2
ATP
ADP
H^+
2.7.1.1/2.7.1.2
D-glucopyranose 6-phosphate
Glycolysis 1 (from glucose 6-phosphate)
B
Rodentibacter pneumotropicus ASV2
malQ
MUU45_000722
MUU45_000723
MUU45_001354
MUU45_001594
C
Rodentibacter heylii ASV5
malQ
glgP
MUW69_001339
MUW69_001340
MUW69_001781
MUU69_001878

Supplemental Figure 7. Heatmap illustrating taxa prominent in the technical controls. ASVs were included if they were ≥ 1% average relative abundance in either swab blank technical controls or tissue blank technical controls. Hierarchical clustering was performed on rows and columns (based on control type) and ASVs identified as contaminants and that were removed from the biological datasets are highlighted in red.

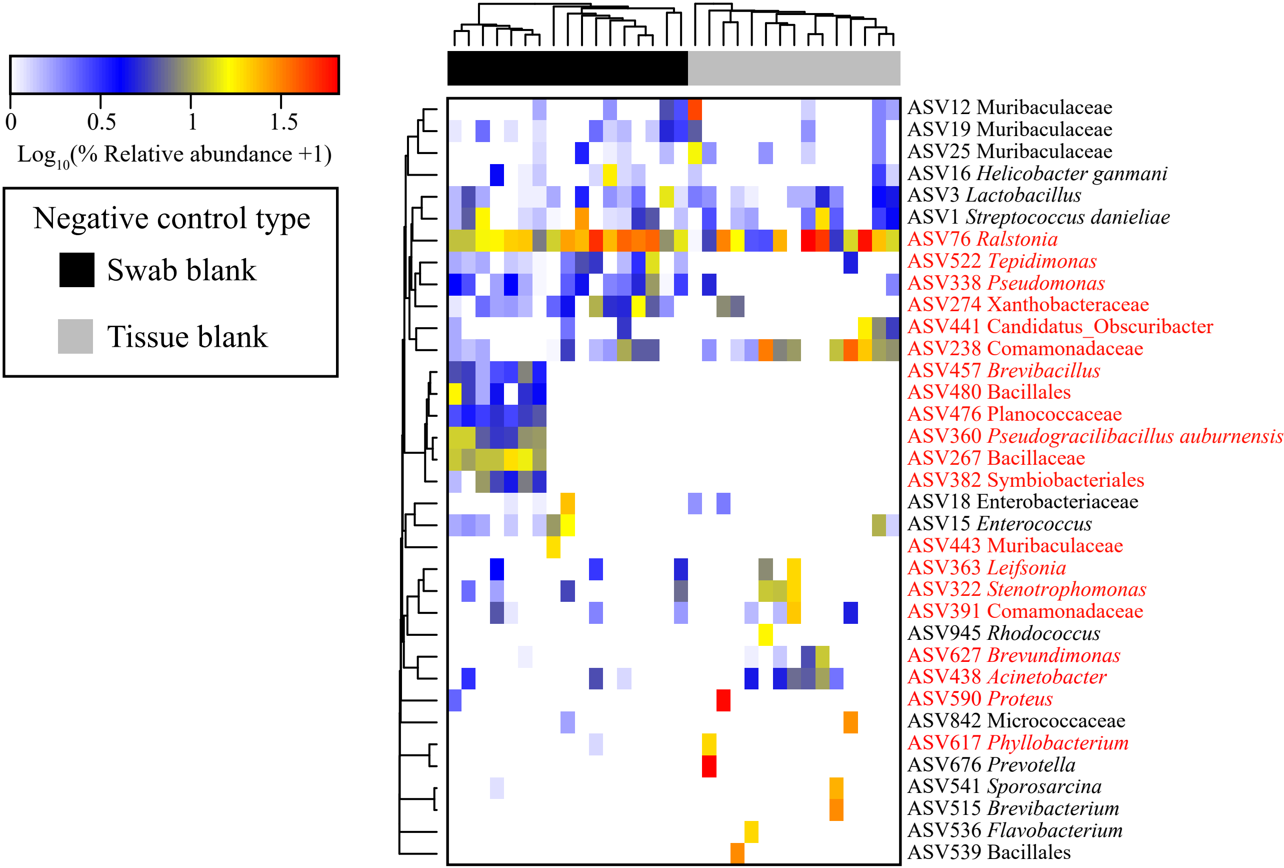

0
0.5
1
1.5
Log10(% Relative abundance +1)
Negative control type
Swab blank
Tissue blank
ASV12 Muribaculaceae
ASV19 Muribaculaceae
ASV25 Muribaculaceae
ASV16 Helicobacter ganmani
ASV3 Lactobacillus
ASV1 Streptococcus danieliae
ASV76 Ralstonia
ASV522 Tepidimonas
ASV338 Pseudomonas
ASV274 Xanthobacteraceae
ASV441 Candidatus_Obscuribacter
ASV238 Comamonadaceae
ASV457 Brevibacillus
ASV480 Bacillales
ASV476 Planococcaceae
ASV360 Pseudogracilibacillus auburnensis
ASV267 Bacillaceae
ASV382 Symbiobacteriales
ASV18 Enterobacteriaceae
ASV15 Enterococcus
ASV443 Muribaculaceae
ASV363 Leifsonia
ASV322 Stenotrophomonas
ASV391 Comamonadaceae
ASV945 Rhodococcus
ASV627 Brevundimonas
ASV438 Acinetobacter
ASV590 Proteus
ASV842 Micrococcaceae
ASV617 Phyllobacterium
ASV676 Prevotella
ASV541 Sporosarcina
ASV515 Brevibacterium
ASV536 Flavobacterium
ASV539 Bacillales

References

1. Abusleme L, Hong B-Y, Hoare A, Konkel JE, Diaz PI, Moutsopoulos NM. 2017. Oral Microbiome Characterization in Murine Models. Bio-protocol 7:e2655.
2. Hernández-Arriaga A, Baumann A, Witte OW, Frahm C, Bergheim I, Camarinha-Silva A. 2019. Changes in Oral Microbial Ecology of C57BL/6 Mice at Different Ages Associated with Sampling Methodology. Microorganisms 7:283.
3. Abusleme L, O'Gorman H, Dutzan N, Greenwell-Wild T, Moutsopoulos NM. 2020. Establishment and Stability of the Murine Oral Microbiome. Journal of Dental Research 99:721-729.
4. Jašarević E, Hill EM, Kane PJ, Rutt L, Gyles T, Folts L, Rock KD, Howard CD, Morrison KE, Ravel J, Bale TL. 2021. The composition of human vaginal microbiota transferred at birth affects offspring health in a mouse model. Nature Communications 12:6289.
5. Barfod KK, Roggenbuck M, Hansen LH, Schjørring S, Larsen ST, Sørensen SJ, Krogfelt KA. 2013. The murine lung microbiome in relation to the intestinal and vaginal bacterial communities. BMC microbiology 13:303-303.
6. Poroyko V, Meng F, Meliton A, Afonyushkin T, Ulanov A, Semenyuk E, Latif O, Tesic V, Birukova AA, Birukov KG. 2015. Alterations of lung microbiota in a mouse model of LPS-induced lung injury. American journal of physiology Lung cellular and molecular physiology 309:L76-L83.
7. Singh N, Vats A, Sharma A, Arora A, Kumar A. 2017. The development of lower respiratory tract microbiome in mice. Microbiome 5:61-61.
8. Dickson RP, Erb-Downward JR, Falkowski NR, Hunter EM, Ashley SL, Huffnagle GB. 2018. The Lung Microbiota of Healthy Mice Are Highly Variable, Cluster by Environment, and Reflect Variation in Baseline Lung Innate Immunity. American journal of respiratory and critical care medicine 198:497-508.
9. Kostric M, Milger K, Krauss-Etschmann S, Engel M, Vestergaard G, Schloter M, Schöler A. 2018. Development of a Stable Lung Microbiome in Healthy Neonatal Mice. Microbial Ecology 75:529-542.
10. Ashley SL, Sjoding MW, Popova AP, Cui TX, Hoostal MJ, Schmidt TM, Branton WR, Dieterle MG, Falkowski NR, Baker JM, Hinkle KJ, Konopka KE, Erb-Downward JR, Huffnagle GB, Dickson RP. 2020. Lung and gut microbiota are altered by hyperoxia and contribute to oxygen-induced lung injury in mice. Science Translational Medicine 12:eaau9959.
11. Li J, Hu Y, Liu L, Wang Q, Zeng J, Chen C. 2020. PM2.5 exposure perturbs lung microbiome and its metabolic profile in mice. Sci Total Environ 721:137432.
12. Baker JM, Hinkle KJ, McDonald RA, Brown CA, Falkowski NR, Huffnagle GB, Dickson RP. 2021. Whole lung tissue is the preferred sampling method for amplicon-based characterization of murine lung microbiota. Microbiome 9:99.
13. Lipinski JH, Falkowski NR, Huffnagle GB, Erb-Downward JR, Dickson RP, Moore BB, O'Dwyer DN. 2021. Toll-like receptors, environmental caging, and lung dysbiosis. American Journal of Physiology-Lung Cellular and Molecular Physiology 321:L404-L415.

14. Oh JE, Kim B-C, Chang D-H, Kwon M, Lee SY, Kang D, Kim JY, Hwang I, Yu J-W, Nakae S, Lee HK. 2016. Dysbiosis-induced IL-33 contributes to impaired antiviral immunity in the genital mucosa. Proceedings of the National Academy of Sciences 113:E762-E771.
15. Vrbanac A, Riestra AM, Coady A, Knight R, Nizet V, Patras KA. 2018. The murine vaginal microbiota and its perturbation by the human pathogen group B Streptococcus. BMC Microbiology 18:197.
16. Karpinets TV, Solley TN, Mikkelson MD, Dorta-Estremera S, Nookala SS, Medrano AYD, Petrosino JF, Mezzari MP, Zhang J, Futreal PA, Sastry KJ, Colbert LE, Klopp A. 2020. Effect of Antibiotics on Gut and Vaginal Microbiomes Associated with Cervical Cancer Development in Mice. Cancer Prevention Research 13:997-1006.
17. Hernández-Quiroz F, Murugesan S, Velazquez-Martínez C, Villalobos-Flores LE, Maya-Lucas O, Piña-Escobedo A, García-González I, Ocadiz-Delgado R, Lambert PF, Gariglio P, García-Mena J. 2021. The vaginal and fecal microbiota of a murine cervical carcinoma model under synergistic effect of 17β-Estradiol and E7 oncogene expression. Microbial Pathogenesis 152:104763.
18. Mejia ME, Ottinger S, Vrbanac A, Babu P, Zulk JJ, Moorshead D, Bode L, Nizet V, Patras KA, Fey PD. 2022. Human Milk Oligosaccharides Reduce Murine Group B *Streptococcus* Vaginal Colonization with Minimal Impact on the Vaginal Microbiota. mSphere 7:e00885-21.
